# Impact on the ability of healthcare professionals to correctly identify patient-ventilator asynchronies of the simultaneous visualization of estimated muscle pressure curves on the ventilator display: a randomized study (*P*_mus_ study)

**DOI:** 10.1186/s13054-023-04414-9

**Published:** 2023-03-30

**Authors:** Daniel Oliveira Silva, Patrícia Nery de Souza, Mayson Laercio de Araujo Sousa, Caio Cesar Araujo Morais, Juliana Carvalho Ferreira, Marcelo Alcantara Holanda, Wellington Pereira Yamaguti, Laerte Pastore Junior, Eduardo Leite Vieira Costa

**Affiliations:** 1 Intensive Care Unit, Hospital Sírio-Libanes, São Paulo, Brazil; 2 Interdepartmental Division of Critical Care Medicine, St. Michael’s Hospital, Toronto, Canada; 3 Universidade Federal de Pernambuco, UFPE, Pernambuco, Brazil; 4 Disciplina de Pneumologia, Heart Institute (Incor), Hospital das Clínicas da Faculdade de Medicina da Universidade de São Paulo, São Paulo, Brazil; 5 Departamento de Medicina Clínica, Universidade Federal do Ceará, Fortaleza, Brazil; 6 Programa de Pós-Graduação de Mestrado em Ciências Médicas, Universidade Federal do Ceará, Fortaleza, Brazil; 7 Research and Education Institute, Hospital Sírio-Libanes, São Paulo, Brazil

**Keywords:** Mechanical ventilation, Artificial ventilation, Interactive ventilatory support, Respiratory diaphragm, Respiratory failure

## Abstract

**Background:**

Patient-ventilator asynchronies are usually detected by visual inspection of ventilator waveforms but with low sensitivity, even when performed by experts in the field. Recently, estimation of the inspiratory muscle pressure (*P*_mus_) waveforms through artificial intelligence algorithm has been proposed (Magnamed**®**, São Paulo, Brazil). We hypothesized that the display of these waveforms could help healthcare providers identify patient-ventilator asynchronies.

**Methods:**

A prospective single-center randomized study with parallel assignment was conducted to assess whether the display of the estimated P_mus_ waveform would improve the correct identification of asynchronies in simulated clinical scenarios. The primary outcome was the mean asynchrony detection rate (sensitivity). Physicians and respiratory therapists who work in intensive care units were randomized to control or intervention group. In both groups, participants analyzed pressure and flow waveforms of 49 different scenarios elaborated using the ASL-5000 lung simulator. In the intervention group the estimated *P*_mus_ waveform was displayed in addition to pressure and flow waveforms.

**Results:**

A total of 98 participants were included, 49 per group. The sensitivity per participant in identifying asynchronies was significantly higher in the *P*_mus_ group (65.8 ± 16.2 vs. 52.94 ± 8.42, *p* < 0.001). This effect remained when stratifying asynchronies by type.

**Conclusions:**

We showed that the display of the *P*_mus_ waveform improved the ability of healthcare professionals to recognize patient-ventilator asynchronies by visual inspection of ventilator tracings. These findings require clinical validation.

*Trial registration*: ClinicalTrials.gov: NTC05144607. Retrospectively registered 3 December 2021.

**Supplementary Information:**

The online version contains supplementary material available at 10.1186/s13054-023-04414-9.

## Background

During assisted modes of mechanical ventilation, patient-ventilator asynchronies can occur because of a mismatch between neural (patient) and ventilator inspiratory and expiratory phases [1]. Patients on mechanical ventilation present some types of asynchrony in up to 40% of respiratory cycles [2–5]. In general, they can be characterized by occurring in situations of excessive ventilatory assistance and/or low respiratory drive and in situations of insufficient ventilatory assistance and/or increased respiratory drive. The occurrence of asynchronies has been associated with longer lengths of mechanical ventilation and even higher mortality rates [6, 7] especially double trigger[8] or ineffective effort[9] when they occur in clusters with high power and of long duration[10, 11]. Resolution of these asynchronies through changes in ventilator settings or other measures, such as sedation, depends on the correct identification of the type of asynchrony. Misdiagnoses can lead to inadequate adjustments of ventilatory parameters resulting in a vicious cycle of sedation, controlled mechanical ventilation, and diaphragmatic dysfunction [3, 5, 12–14].

Typically, asynchronies are detected at the bedside by healthcare professionals by visual inspection of ventilator waveforms [1, 15]. However, the sensitivity of this visual analysis is low even when performed by experts in the field, amounting to less than a third of asynchronous respiratory cycles [16]. To improve this sensitivity, the display of the electrical activity of the diaphragm (Eadi) or of the esophageal or transdiaphragmatic pressure signal has been proposed as a way to enhance asynchrony detection [17–21]. However, these techniques require the insertion of esophageal catheters, which is invasive and can be technically challenging.

An alternative way to obtain inspiratory muscle pressure (P_mus_) estimations has been recently proposed (Magnamed®, São Paulo, Brazil). Through artificial intelligence, a proprietary algorithm receives pressure, flow, and volume as inputs and returns the estimated P_mus_ waveform on the ventilator screen (for further details, see online supplement). The display of P_mus_ could help healthcare providers to assess whether the start and end of the mechanical breath are in synchrony with the patient effort as visualized in the P_mus_ waveform. In the present study, we aimed to test the hypothesis that the visualization of P_mus_ together with the other ventilator waveforms on the ventilator display would improve the ability of healthcare professionals to identify asynchronies.

## Material and methods

### Study design and setting

This is a prospective single-center randomized study with parallel assignment conducted at the Research and Education Institute (IEP) of the Sírio Libanês Hospital (São Paulo, Brazil).

### Study participants

Physicians and respiratory therapists who worked in one of the eight mixed medical/surgical intensive care units of the Hospital Sírio Libanês, São Paulo, Brazil, were invited via email to participate in the study. All participants were experienced with the bedside detection of asynchronies by visual inspection of ventilator waveforms.

### Randomization

Individuals who agreed to participate were randomized on a 1:1 ratio, stratified by time of experience and profession, to the control or the intervention group (*P*_mus_ group). Randomization was performed using a computer-generated random list. Participants remained unaware of the group to which they were assigned until the session began.

### Interventions

Before randomization, participants watched a 30-min online refresh course on asynchronies definitions based on previously published criteria [1, 22] using the Zoom® platform (Zoom Video Communications, California, USA). All the waveforms presented in the course were obtained from the literature. After the presentation, participants could interact with instructors in a questions and answers session.

After this run-in phase, participants were randomized to either the control or the *P*_mus_ group. None of the participants had previous experience with the ventilator used for the simulations or with the display of *P*_mus_ waveforms estimated with artificial intelligence. Both groups were exposed to identical recordings of simulated scenarios containing asynchronies or synchronous cycles generated using the ASL-5000 active breathing simulator (Ingmar Medical, Pittsburg, PA) connected to the ventilator FlexiMag Max 700 (Magnamed, São Paulo, Brazil).

A total of 49 scenarios were elaborated including synchronous cycles and the following asynchronies: ineffective effort, auto-triggering, double-triggering, reverse triggering, reverse triggering with double cycling, premature cycling, and late cycling. The scenarios were created using different conditions of respiratory system mechanics and patient effort in accordance with standard asynchronies definitions published in the literature (Table 1) [1, 22]. The asynchronies were classified using the patient effort programmed in the lung simulator ASL 5000, which was considered the gold standard *P*_mus_. The *P*_mus_ waveform displayed on the ventilator was estimated with a machine learning algorithm embedded in the ventilator FlexiMag Max 700 and based on proprietary software (Magnamed, São Paulo, Brazil). The algorithm uses a recurrent neural network called Long Short-Term Memory (LSTM) to estimate *P*_mus_ from airway pressure, flow, and volume (for further details, see Additional file 1: eFigure1) and has been validated against simulated data (for further details, see Additional file 1: eFigures 2–4). The software is not yet approved for clinical use. A clinical validation study is ongoing.

**Table 1:** Definitions of the types of asynchronies

Types of asynchronies	Definition
Double-triggering	Two ventilator cycles triggered by a single effort
Ineffective effort	Presence of effort (*P*_mus_) without ventilator triggering
Premature cycling	Inspiratory time too short compared to the patient, defined as cycling to the expiratory phase before peak *P*_mus_
Delayed cycling	Inspiratory time too long in relation to the patient: defined as cycling to the expiratory phase after the end of the effort (*P*_mus_)
Reverse triggering	*P*_mus_ follows the controlled (or auto-triggered) cycle with a fixed frequency and delay. May or may not generate double cycle
Reverse triggering with double cycling	*P*_mus_ follows the controlled (or auto-triggered) cycle with a fixed frequency and delay. May or may not generate double cycle
Auto-triggering	Nonpatient effort (*P*_mus_) with ventilator triggering

*P*_mus_ inspiratory muscle pressure

Each scenario contained a 30-s recording followed by 30 s of a still ventilator screen. During this one-minute period, participants were instructed to choose whether they identified asynchronies and which asynchrony was present using the voting tool Mentimeter (Mentimeter, Sweden).

**Fig. 1:**
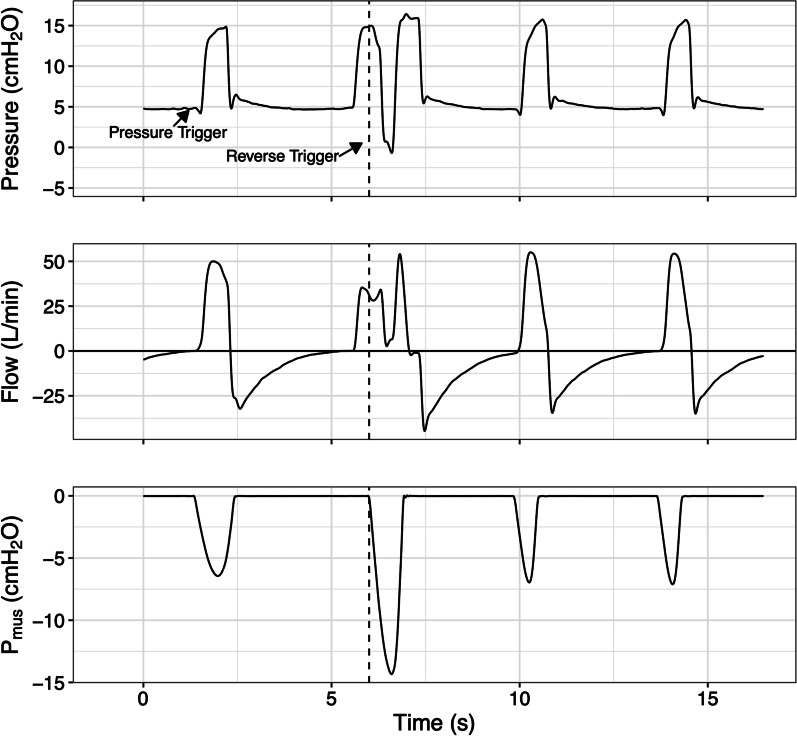
Schematic example of waveforms from one of the simulated scenarios. The tracings represent the airway pressure, flow, and estimated inspiratory muscle pressure (*P*_mus_). Note that the second ventilatory cycle is controlled and that the effort starts during the ventilator inspiratory phase, simulating a reverse triggering event. The *P*_mus_ waveform with the vertical line indicating the start of effort was available only to the *P*_mus_ group

For the control group, the recordings showed conventional pressure and flow waveforms over time (Additional file 1: eTable1). For the *P*_mus_ group, using the same simulated scenarios and in the same order, the waveform of estimated *P*_mus_ over time was also displayed, in addition to the pressure and flow waveforms (Fig. 1 and Additional file 1: eFigures 5–12).

### Study endpoints

The mean asynchrony detection rate (sensitivity) was the primary endpoint, and specificity was a secondary endpoint. Sensitivity refers to the probability of correctly identifying an asynchrony, and specificity was defined as the probability of correctly identifying the absence of asynchronies. Both probabilities were calculated for each participant for all asynchronies together and according to asynchrony type considering the answer key.

### Sample size estimation

Based on a previous study [16] in which participants had an average sensitivity of 28% to detect asynchronies, we estimated that the inclusion of 98 participants would have 90% power to detect a 10-percentage-point difference in the mean sensitivity between groups with a two-tailed significance level of 0.05 assuming the standard deviation to be 15 percentage points.

### Data analysis

Deidentified participants' responses were stored and subsequently compared against the answer key. For each participant, sensitivity and specificity were calculated considering all asynchronies together and then again according to asynchrony type. The means of these variables were compared between the control and *P*_mus_ groups.

Data normality was verified by the Shapiro–Wilk test. Variables with normal distribution were described using mean and standard deviation and compared using the Student’s t test, while variables with non-normal distribution were described as median and interquartile range and compared using the Mann–Whitney test.

A *p* < 0.05 was considered significant. Statistical analysis was performed using R (version 3.5.2).

## Results

A total of 98 participants were included, 49 per group. Most participants had more than 5 years’ experience (65.3% in the control group vs. 67.4% in the *P*_mus_ group). Groups were also balanced regarding profession (physicians 25.45% in the control group vs. 23.07% in the *P*_mus_ group, and respiratory therapists 74.55% in the control group vs. 76.93% in the *P*_mus_ group).

**Fig. 2:**
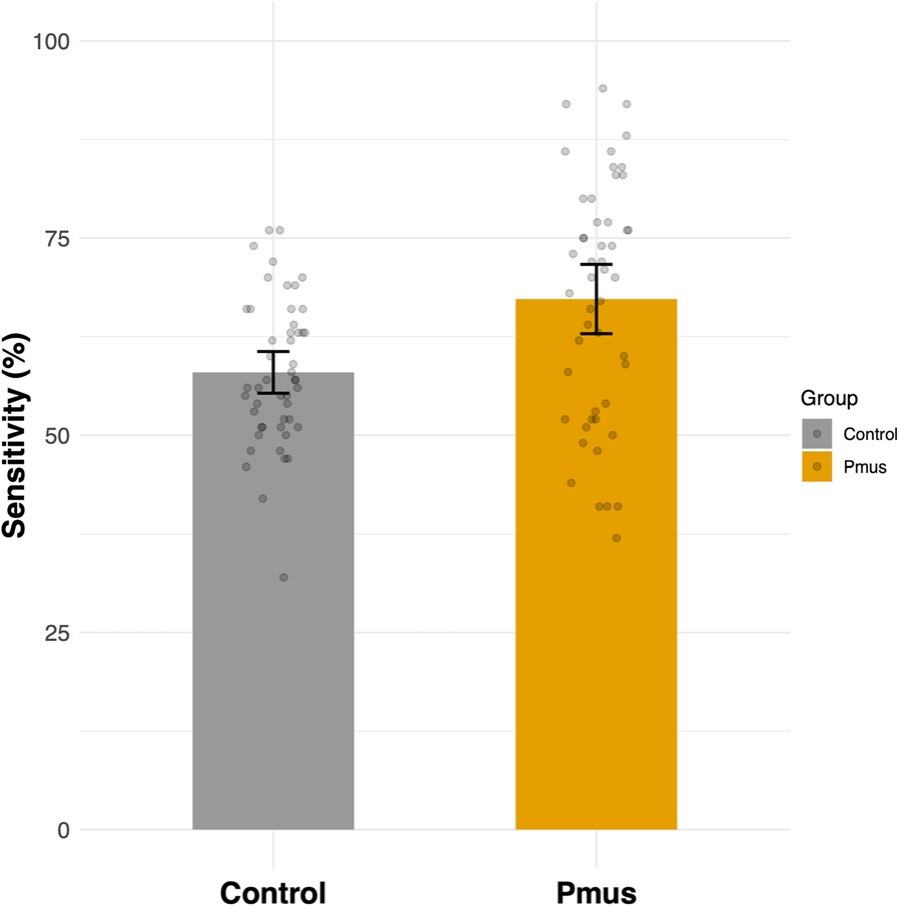
Asynchrony detection rate (sensitivity) in the inspiratory muscle pressure (*P*_mus_) group as compared to the control group. Error bars represent the standard error of the mean, and dots represent the individual sensitivity per participant

Mean sensitivity was higher in the *P*_mus_ group as compared to the control group (65.8 ± 16.2 vs 52.94 ± 8.42%, *p* < 0.001) (Fig. 2). This effect was observed also when we considered asynchronies by type (Additional file 1: eFigure 13). On the other hand, there was no difference between the groups in the identification of synchronous curves. The mean specificity per participant was similar between groups independently of asynchrony type (Additional file 1: eFigure 14).

## Discussion

We found that the addition of the estimated *P*_mus_ to the pressure and flow waveforms increased the sensitivity of respiratory therapists and physicians to identify various types of asynchronies without affecting their specificity. Asynchrony detection rate in the control group was just over 50% and increased by approximately 20% in the *P*_mus_ group.

Despite all advances in mechanical ventilation, patient-ventilator asynchrony is still common [2–5] and its detection remains a challenge [16, 23–25]. Undoubtedly, there is a gap in knowledge among healthcare professionals that hinders the correct identification of asynchronies [16, 25–27]. However, even experts in the field have difficulty detecting asynchronous cycles, with sensitivity values reported as low as 28% [16]. Monitoring of esophageal pressure or of electrical activity of the diaphragm can improve the detection of asynchronies, but those monitoring techniques are invasive, costly, and require specific equipment [17, 19, 20]. Automated detection of asynchronies based on ventilator waveform analysis has also been proposed, such as the Better Care ® [28] but has not been incorporated into clinical practice. Recently a pilot study proposed the use of a machine learning (ML) algorithm to replicate human expertise in detecting patient-ventilator cycling asynchrony based on waveform analysis with a strong agreement [29]. Although of value, these efforts to replicate human expertise have the inherent limitation of the low performance of humans to detect asynchronies with only conventional ventilator waveforms. Ge H et al. applied ML to identify patient-ventilator asynchrony offline using big data in a retrospective study. The results corroborate the importance to recognize asynchronies and to use ML for this purpose in clinical practice [30].

In the current study, we took the approach to noninvasively estimate the *P*_mus_ waveform based on artificial intelligence and used that information together with conventional ventilator waveform. At the conceptual level, this approach is equivalent to having the *P*_mus_ obtained from esophageal pressures monitoring but without the invasiveness and technical challenges of the esophageal balloon placement. We confirmed our hypothesis that the display of this additional waveform facilitated asynchronies detection.

Our finding of a 20% increase in the detection rate of asynchronies corresponded to an absolute increase of 13 percentage points. Conversely, specificity was high in the control group and was not affected by the intervention likely because health professionals seldom overdiagnose asynchronies. We believe that this modest increase in the asynchronies’ detection rate was at least in part related to the fact that participants had no previous training with visualization of the *P*_mus_ and thus no experience relating that waveform to pressure and flow. If that is the case, it is possible that the diagnostic performance increases with practice suggesting that future studies should include a run-in phase consisting of training sessions. For example, the detection rate of auto-triggering was still less than 60% in the intervention group when it should have been easy to the trained eye to identify that the cycle was not accompanied by muscle effort (Additional file 1: eFigure 9). Furthermore, incorporating visual cues in the ventilator display to indicate the phases of the patient's effort during the respiratory cycle, in addition to simply incorporating the *P*_mus_ waveform, could facilitate the detection of asynchronies.

Our study has some strengths and limitations. In the run-in period, all participants had access to a lecture with the goal to uniformize their definitions of the various asynchronies according to the current literature [1, 22]. Although attendance to this lecture was not obligatory, more than 90% of participants participated. Our randomized design was important to balance participants in both groups. Considering that expertise can affect the sensitivity to identify patient–ventilator asynchronies through ventilator waveforms [16], we took the additional precaution to randomize our groups stratified by experience and profession. Both study groups were exposed to the exact same waveforms, which helped isolate the effect of the display of the *P*_mus_ curve on the asynchrony detection rate. Among the limitations of the study, some stand out. First, all scenarios were obtained using the ASL-5000 active breathing simulator, not real ventilator tracings recorded from patients. Simulated efforts are stereotyped and easier to interpret when compared to the chaotic effort pattern sometimes seen in the real world. To minimize this limitation, we designed scenarios that simultaneously incorporated more than one type of asynchrony to better reflect the diversity seen in clinical settings. Second, 49 scenarios cannot represent all variations of the different asynchronies. Third, participants did not have the chance to familiarize themselves with the use of the *P*_mus_ curve. Fourth, the estimated *P*_mus_ curve still lacks clinical validation. Consequently, the results only prove that asynchrony detection has the potential to improve if the *P*_mus_ estimation is proved acceptable in the clinical scenario. Finally, this was a proof-of-concept, single-center study and requires validation, because knowledge and practices regarding asynchronies can vary by center.

## Conclusion

Using simulated scenarios, we showed that the display of estimated *P*_mus_ waveform improved the ability of healthcare professionals to recognize patient–ventilator asynchronies by visual inspection of ventilator tracings. Further studies should be undertaken to verify the validity of our findings in the clinical setting.

## Supplementary Information

**Additional file 1.** Supplementary material.

## Data Availability

The datasets used and/or analyzed during the current study are available from the corresponding author on reasonable request.

## Abbreviations

P_mus_: Inspiratory muscle pressure
Eadi: Electrical activity of the diaphragm
C_cw_: Compliance of the chest wall

## References

[1] Dres M, Rittayamai N, Brochard L (2016). Monitoring patient-ventilator asynchrony. Curr Opin Crit Care.

[2] Pohlman MC, McCallister KE, Schweickert WD, Pohlman AS, Nigos CP, Krishnan JA (2008). Excessive tidal volume from breath stacking during lung-protective ventilation for acute lung injury. Crit Care Med.

[3] Sottile PD, Albers D, Smith BJ, Moss MM (2020). Ventilator dyssynchrony—detection, pathophysiology, and clinical relevance: a Narrative review. Ann Thorac Med.

[4] Mellott KG, Grap MJ, Munro CL, Sessler CN, Wetzel PA, Nilsestuen JO (2014). Patient ventilator asynchrony in critically ill adults: frequency and types. Heart Lung.

[5] Pham T, Telias I, Piraino T, Yoshida T, Brochard LJ (2018). Asynchrony consequences and management. Crit Care Clin.

[6] de Wit M, Miller KB, Green DA, Ostman HE, Gennings C, Epstein SK (2009). Ineffective triggering predicts increased duration of mechanical ventilation. Crit Care Med.

[7] Blanch L, Villagra A, Sales B, Montanya J, Lucangelo U, Luján M (2015). Asynchronies during mechanical ventilation are associated with mortality. Intensive Care Med.

[8] Sousa MLEA, Magrans R, Hayashi FK, Blanch L, Kacmarek RM, Ferreira JC (2021). Clusters of double triggering impact clinical outcomes: insights from the epidemiology of patient-ventilator asynchrony (EPISYNC) cohort study. Crit Care Med.

[9] Vaporidi K, Babalis D, Chytas A, Lilitsis E, Kondili E, Amargianitakis V (2017). Clusters of ineffective efforts during mechanical ventilation: impact on outcome. Intensive Care Med.

[10] de Araújo Sousa ML, Magrans R, Hayashi FK, Blanch L, Kacmarek RM, Ferreira JC (2020). Predictors of asynchronies during assisted ventilation and its impact on clinical outcomes: the EPISYNC cohort study. J Crit Care.

[11] Magrans R, Ferreira F, Sarlabous L, López-Aguilar J, Gomà G, Fernandez-Gonzalo S (2022). The effect of clusters of double triggering and ineffective efforts in critically Ill patients. Crit Care Med.

[12] Schreiber A, Bertoni M, Goligher EC (2018). Avoiding respiratory and peripheral muscle injury during mechanical ventilation: diaphragm-protective ventilation and early mobilization. Crit Care Clin.

[13] Goligher EC, Brochard LJ, Reid WD, Fan E, Saarela O, Slutsky AS (2019). Diaphragmatic myotrauma: a mediator of prolonged ventilation and poor patient outcomes in acute respiratory failure. Lancet Respir Med.

[14] Gilstrap D, MacIntyre N (2013). Patient-ventilator interactions. Implications for clinical management. Am J Respir Crit Care Med.

[15] Georgopoulos D, Prinianakis G, Kondili E (2006). Bedside waveforms interpretation as a tool to identify patient-ventilator asynchronies. Intensive Care Med.

[16] Colombo D, Cammarota G, Alemani M, Carenzo L, Barra FL, Vaschetto R (2011). Efficacy of ventilator waveforms observation in detecting patient-ventilator asynchrony. Crit Care Med.

[17] Kondili E, Prinianakis G, Georgopoulos D (2003). Patient-ventilator interaction. Br J Anaesth.

[18] Mauri T, Yoshida T, Bellani G, Goligher EC, Carteaux G, Rittayamai N (2016). Esophageal and transpulmonary pressure in the clinical setting: meaning, usefulness and perspectives. Intensive Care Med.

[19] Akoumianaki E, Maggiore SM, Valenza F, Bellani G, Jubran A, Loring SH (2014). The application of esophageal pressure measurement in patients with respiratory failure. Am J Respir Crit Care Med.

[20] Parthasarathy S, Jubran A, Tobin MJ (2000). Assessment of neural inspiratory time in ventilator-supported patients. Am J Respir Crit Care Med.

[21] Bellani G, Mauri T, Coppadoro A, Grasselli G, Patroniti N, Spadaro S (2013). Estimation of patient’s inspiratory effort from the electrical activity of the diaphragm. Crit Care Med.

[22] Chatburn RL, Mireles-Cabodevila E (2020). 2019 year in review: patient-ventilator synchrony. Respir Care.

[23] Mojoli F, Pozzi M, Orlando A, Bianchi IM, Arisi E, Iotti GA (2022). Timing of inspiratory muscle activity detected from airway pressure and flow during pressure support ventilation: the waveform method. Crit Care.

[24] Thille AW, Rodriguez P, Cabello B, Lellouche F, Brochard L (2006). Patient-ventilator asynchrony during assisted mechanical ventilation. Intensive Care Med.

[25] Murias G, Lucangelo U, Blanch L (2016). Patient-ventilator asynchrony. Curr Opin Crit Care.

[26] Chacón E, Estruga A, Murias G, Sales B, Montanya J, Lucangelo U (2012). Nurses’ detection of ineffective inspiratory efforts during mechanical ventilation. Am J Crit Care.

[27] Ramirez II, Arellano DH, Adasme RS, Landeros JM, Salinas FA, Vargas AG (2017). Ability of ICU health-care professionals to identify patient-ventilator asynchrony using waveform analysis. Respir Care.

[28] Blanch L, Sales B, Montanya J, Lucangelo U, Garcia-Esquirol O, Villagra A (2012). Validation of the better care® system to detect ineffective efforts during expiration in mechanically ventilated patients: a pilot study. Intensive Care Med.

[29] Gholami B, Phan TS, Haddad WM, Cason A, Mullis J, Price L (2018). Replicating human expertise of mechanical ventilation waveform analysis in detecting patient-ventilator cycling asynchrony using machine learning. Comput Biol Med.

[30] Ge H, Duan K, Wang J, Jiang L, Zhang L, Zhou Y (2020). Risk factors for patient-ventilator asynchrony and its impact on clinical outcomes: analytics based on deep learning algorithm. Front Med (Lausanne)..

