# Supplementary material for: Impact on the ability of healthcare professionals to correctly identify patient-ventilator asynchronies of the simultaneous visualization of estimated muscle pressure curves on the ventilator display: a randomized study (Pmus study)
Source: Crit Care. 2023 Mar 30;27:128. doi: 10.1186/s13054-023-04414-9 (PMC10064577; doi:10.1186/s13054-023-04414-9)
Supplement: Supplementary file 1 — Additional file 1. Supplementary material. [file 13054_2023_4414_MOESM1_ESM.docx]

**Additional File 1**

[1. Artificial Intelligence (Machine Learning) Model Architecture 2](#_Toc126936148)

[eFigure 1: 3](#_Toc126936149)

[2. P_mus_ Estimation Performance 4](#_Toc126936150)

[Key performance indices (KPIs) 4](#_Toc126936151)

[eFigure 2 : 4](#_Toc126936152)

[eFigure 3.a : 5](#_Toc126936153)

[eFigure 3.b: 6](#_Toc126936154)

[3. Representative waveforms for illustrative purposes 7](#_Toc126936155)

[eFigure4: 7](#_Toc126936156)

[4. Scenarios of Simulated Asynchronies 8](#_Toc126936157)

[eFigure 5 9](#_Toc126936158)

[eFigure 6 10](#_Toc126936159)

[eFigure 7 11](#_Toc126936160)

[eFigure 8 12](#_Toc126936161)

[eFigure 9 13](#_Toc126936162)

[eFigure 10 14](#_Toc126936163)

[eFigure 11 15](#_Toc126936164)

[eFigure 12 16](#_Toc126936165)

[eTable 1. 17](#_Toc126936166)

[eFigure 13: 23](#_Toc126936167)

[eFigure 14: 24](#_Toc126936168)

[References 25](#_Toc126936169)

# Artificial Intelligence (Machine Learning) Model Architecture

The machine learning algorithm uses input data in the form of airway pressure, flow, and volume from the ventilator to estimate the muscle pressure (P_mus_) waveform. To process the complete sequence of data, a Long Short-Term Memory (LSTM) mode[1-5], which is a subclass of Recurrent Neural Networks (RNN), was developed (as shown in eFigure 1). The model was trained with simulated data for supervised learning in order to establish a relationship between the P_mus_ waveform and the input signal. The training process was interactive: in each training cycle, the structure of the model was updated so that the best fit of the sampled data was obtained. Finally, the last updated model of the training data was validated against simulated data using ASL-5000 active breathing simulator (Ingmar Medical, Pittsburg, PA). The LSTM model was developed in Python using TensorFlow with Keras as the backend library. The specifications of the workstation used for training the models consisted of AMD Ryzen Threadripper 1920X 12-core Processor CPU, 64GB memory and a NVIDIA Geforce RTX 2070 SUPER GPU.

| eFigure 1: A Long Short-Term Memory algorithm was trained to learn muscle pressure curves (P_mus_) from waveforms of airway pressure, flow, and volume during tens of thousands of hours of ventilation. The trained algorithm was used to estimate P_mus_ in the 49 different scenarios of synchronous and asynchronous cycles. | |
| --- | --- |
| \| 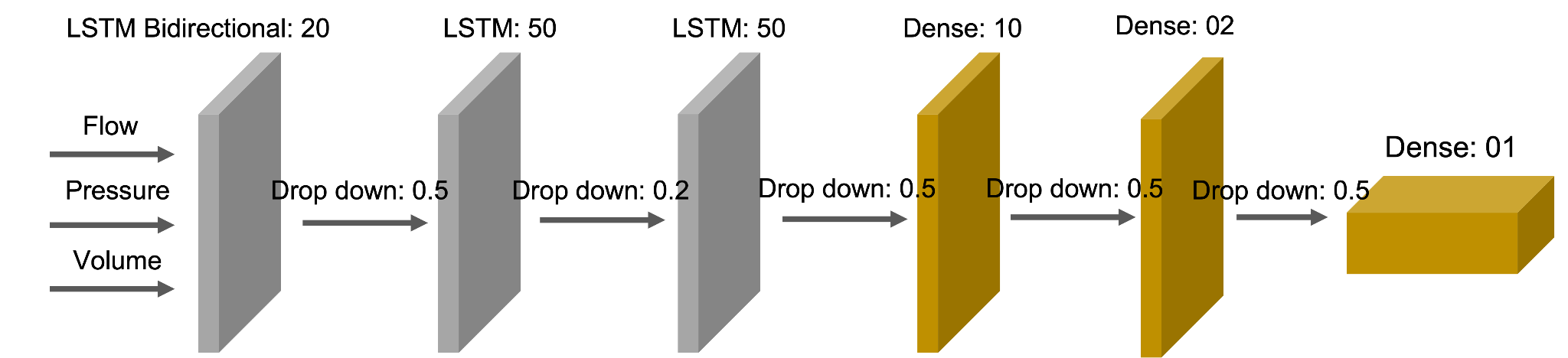 \| \| --- \| |  |

# P_mus_ Estimation Performance

The trained P_mus_ algorithm was tested in a dataset comprised of 23609 respiratory cycles not included in the training set.

## Key performance indices (KPIs)

For the whole test dataset, the difference between simulated (real) P_mus_ and estimated P_mus_ were segmented to identify the following parameters ():

- Start of effort.
- Peak of effort.
- End of effort.
- Amplitude of effort.

| eFigure 2 : KPIs measure |
| --- |
| 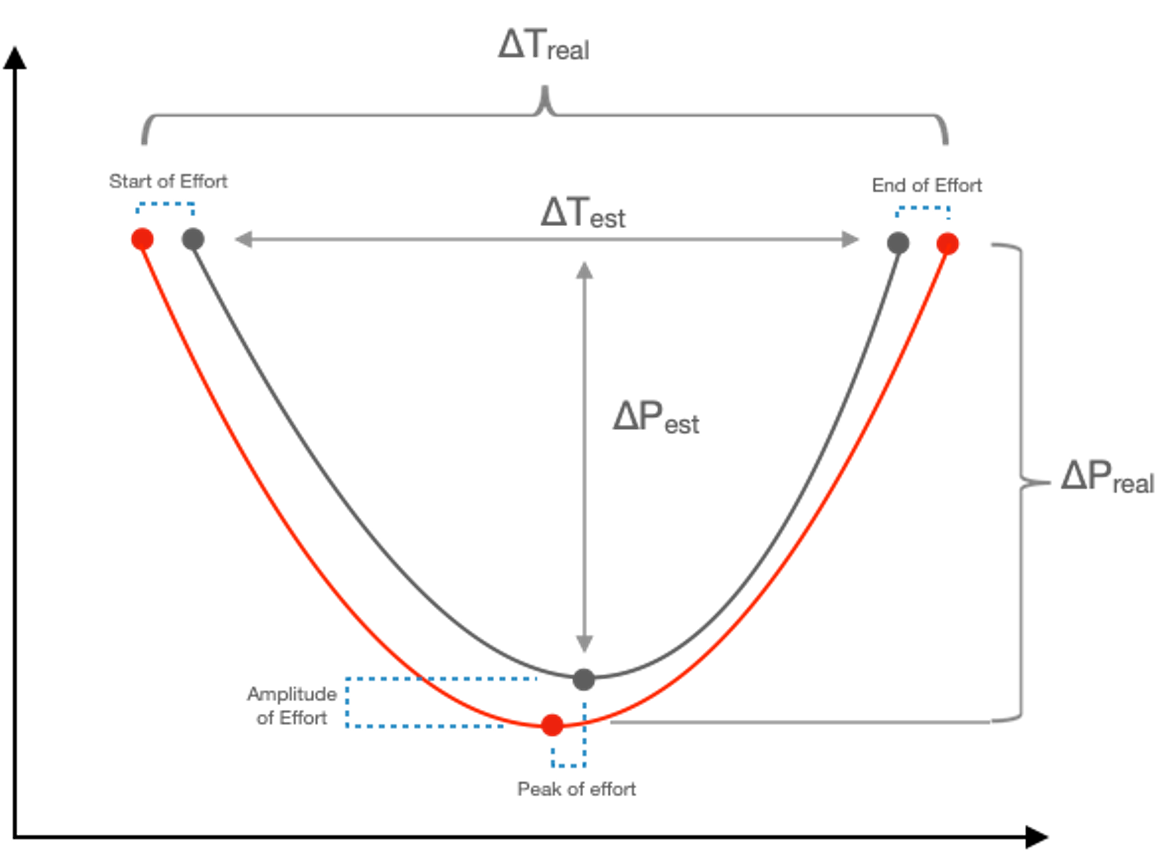 |
| *T _real_* Real time_,_ *T_est_* Estimated time, *P _rea l_* Real pressure and *P_est_* Estimated pressure. |

Errors in the start, peak, and end of effort were considered acceptable if ≤ 100ms. Errors in amplitude were considered acceptable if ≤ 2 cmH_2_O.

| eFigure 3.a : KPIs related to start, peak and end of effort. |
| --- |
| 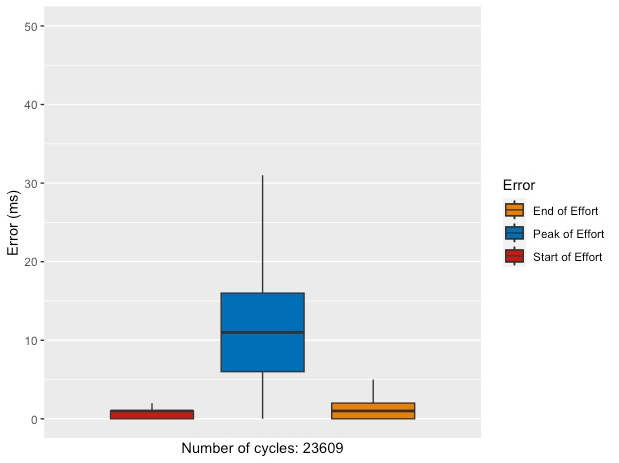 |

| eFigure 3.b: KPI related to amplitude of effort. |
| --- |
| 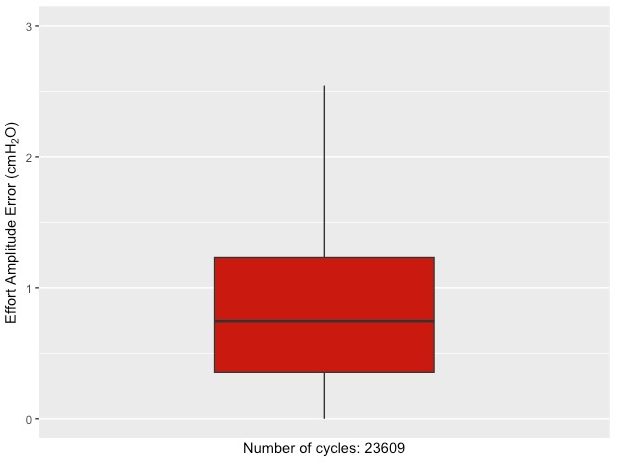 |

# Representative waveforms for illustrative purposes

For illustrative purposes, we show examples of pressure, flow, and P_mus_ waveforms (both estimated and real) in conditions representative of low, high, short, and long efforts (eFigure 4).

|  |  |
| --- | --- |
| eFigure4: Representative waveforms |  |
|  |  |
|  |  |

# Scenarios of Simulated Asynchronies

We selected representative examples of all the asynchronies simulated: reverse trigger, double trigger, ineffective effort, auto-triggering, early cycling, and delayed cycling (eFigures 5-12)

| eFigure 5**:** Example of reverse trigger asynchrony causing double cycling. Identical pressure (green) and flow (blue) waveforms were displayed for both groups. In addition to the conventional waveforms, the P_mus_ group could see the estimated inspiratory muscle pressure (yellow) immediately below the airway pressure (pink). Note that there was an inspiratory effort during the inspiratory phase of the second ventilator cycle, which was controlled. This effort was long enough to trigger a second cycle leading to a reverse triggering asynchrony. | |
| --- | --- |
| 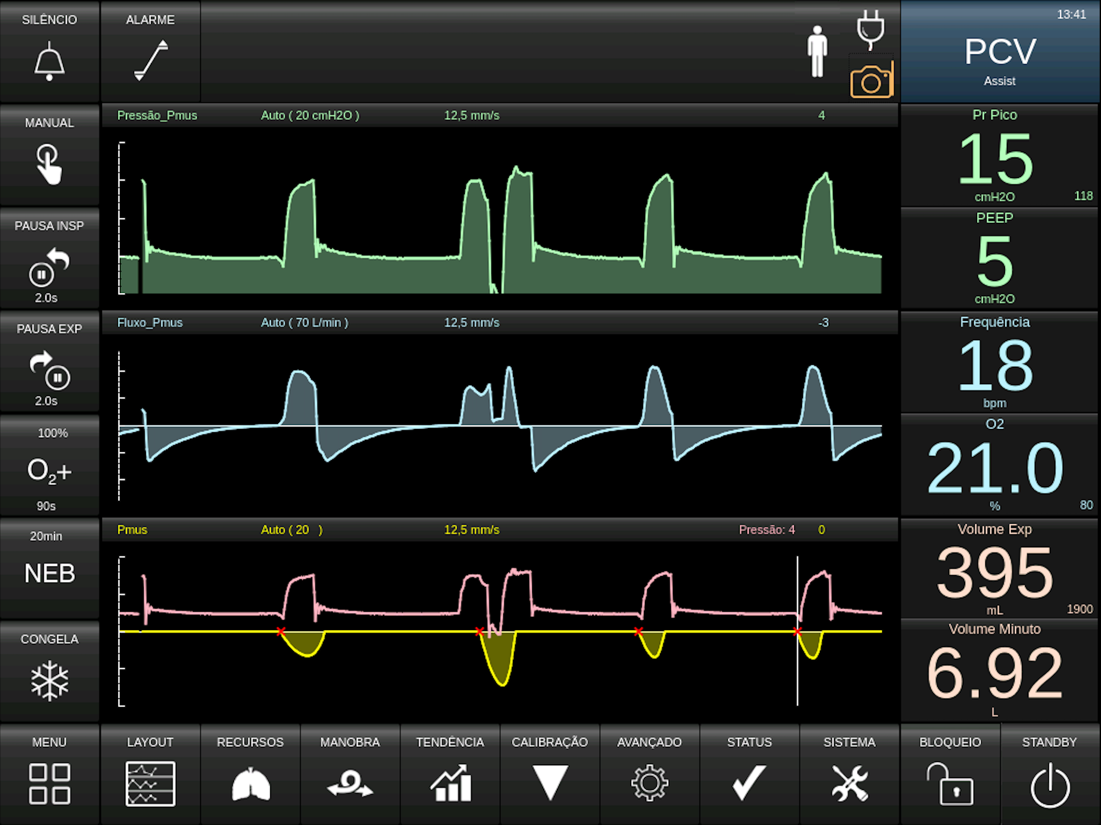  **P_mus_** | 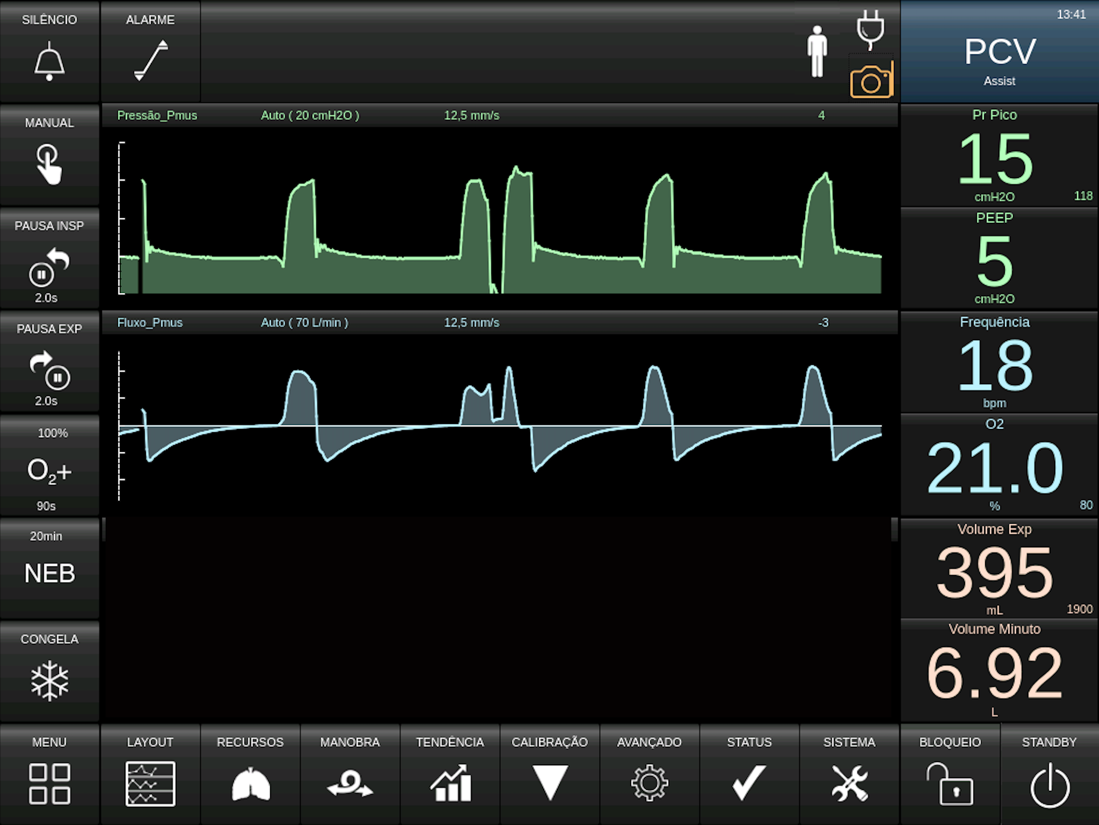  **Control** |

| \| eFigure 6**:** Example of reverse trigger asynchrony without double cycling. Identical pressure (green) and flow (blue) waveforms were displayed for both groups. In addition to the conventional waveforms, the P_mus_ group could see the estimated inspiratory muscle pressure (yellow) immediately below the airway pressure (pink). Note that the first and third ventilator cycles were both controlled and that an effort started during the inspiratory phase causing the already decreasing inspiratory flow to increase again towards the end of inspiration (arrows). \| \| \| --- \| --- \| \| 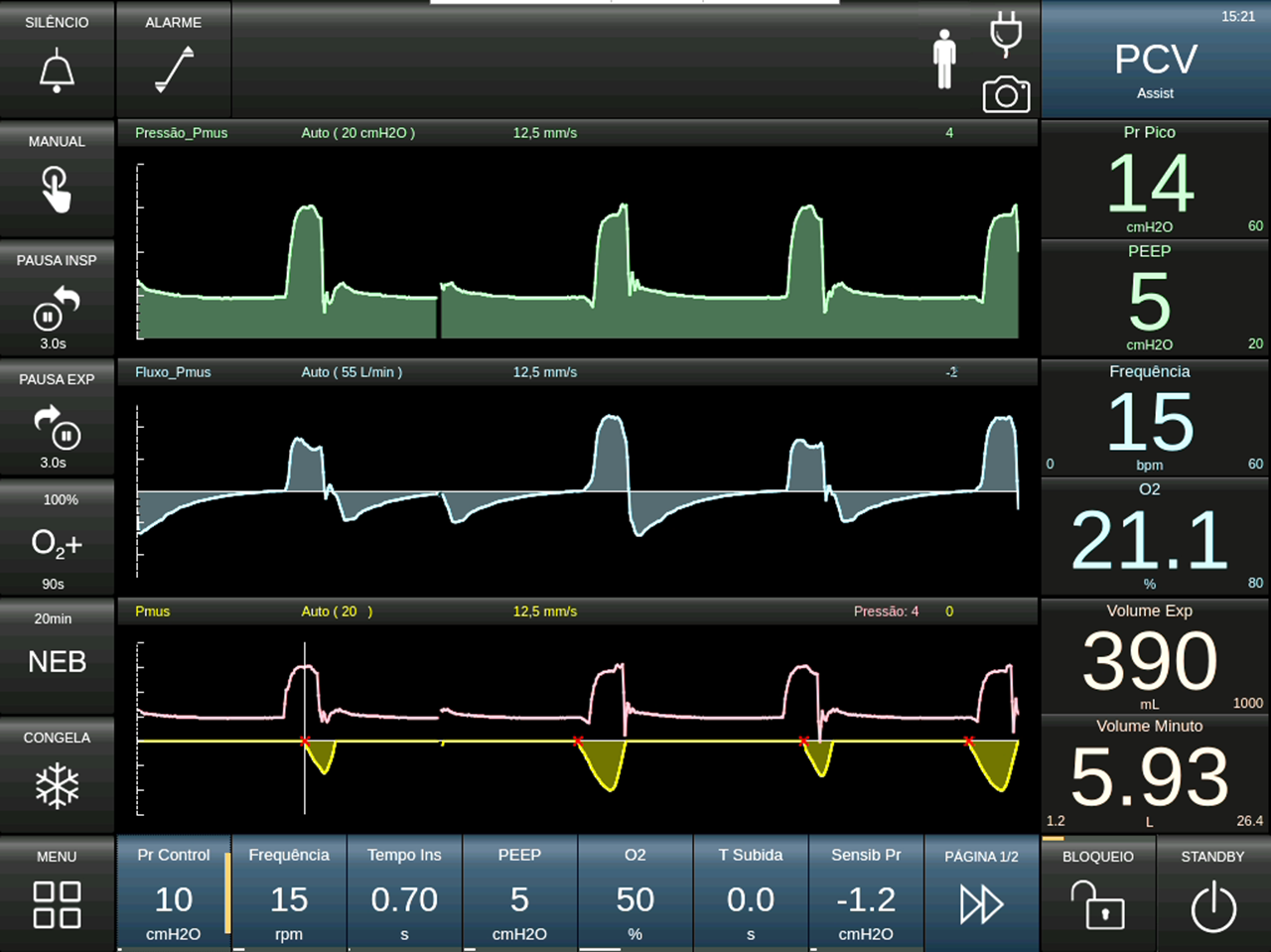  **P_mus_** \| 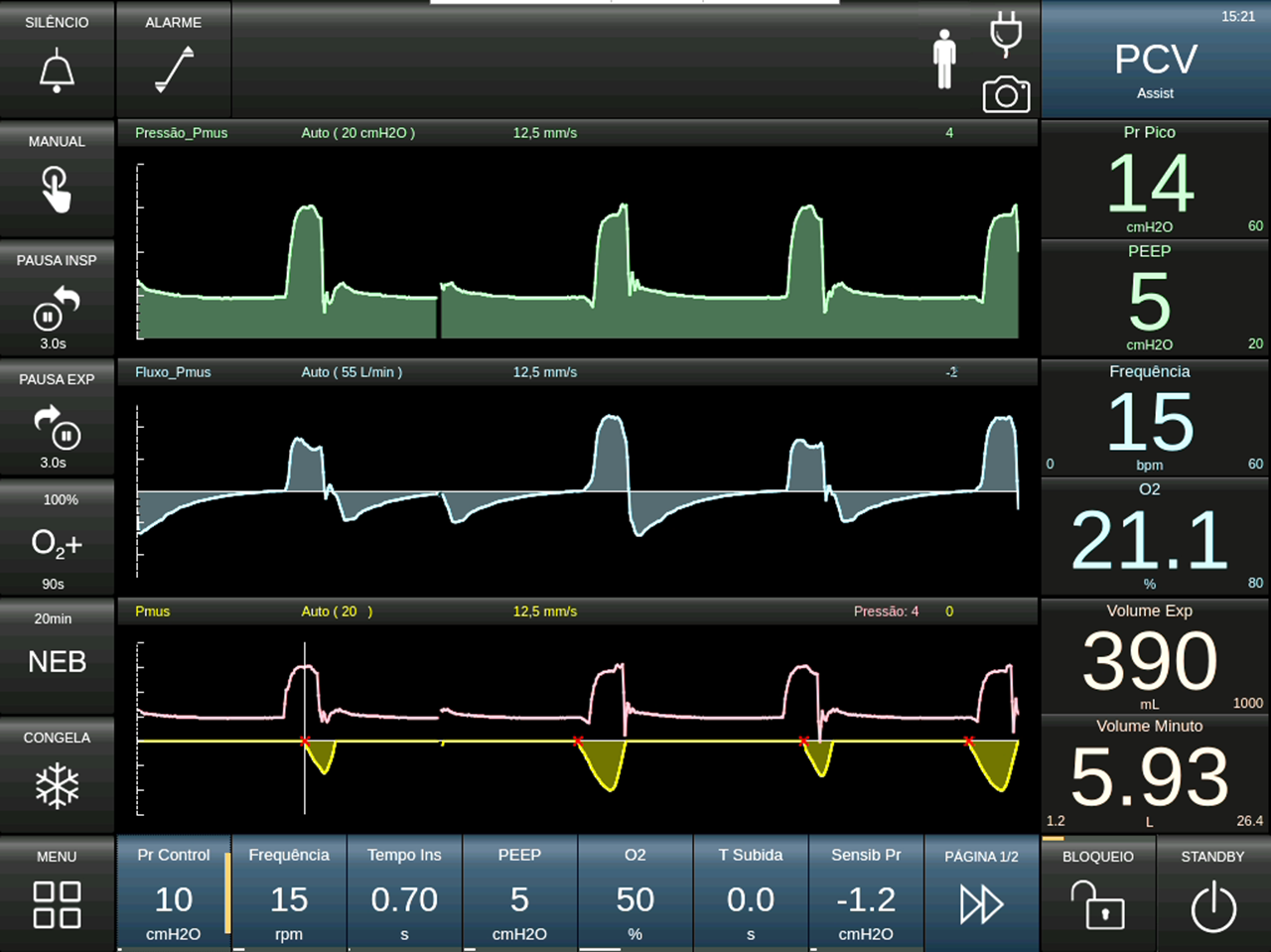  **Control** \|  \| eFigure 7**:** Example of double trigger asynchrony. Identical pressure (green) and flow (blue) waveforms were displayed for both groups. In addition to the conventional waveforms, the P_mus_ group could see the estimated inspiratory muscle pressure (yellow) immediately below the airway pressure (pink). Note that the second and third ventilator cycles were both triggered by the same strong and prolonged effort, thus consisting of a double trigger. The same phenomenon occurred in the fourth and fifth cycles. \| \| \| --- \| --- \| \| 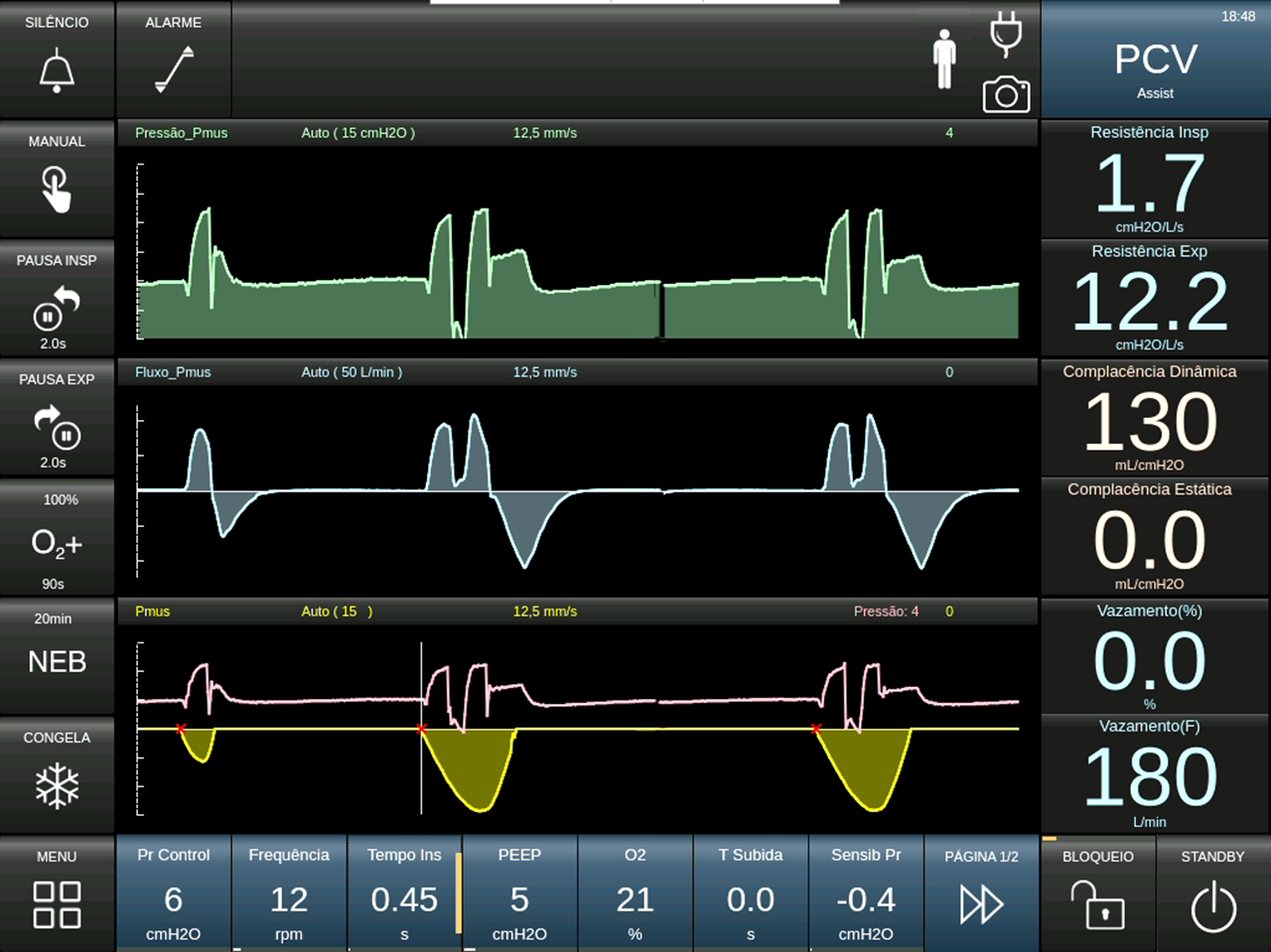  **P_mus_** \| 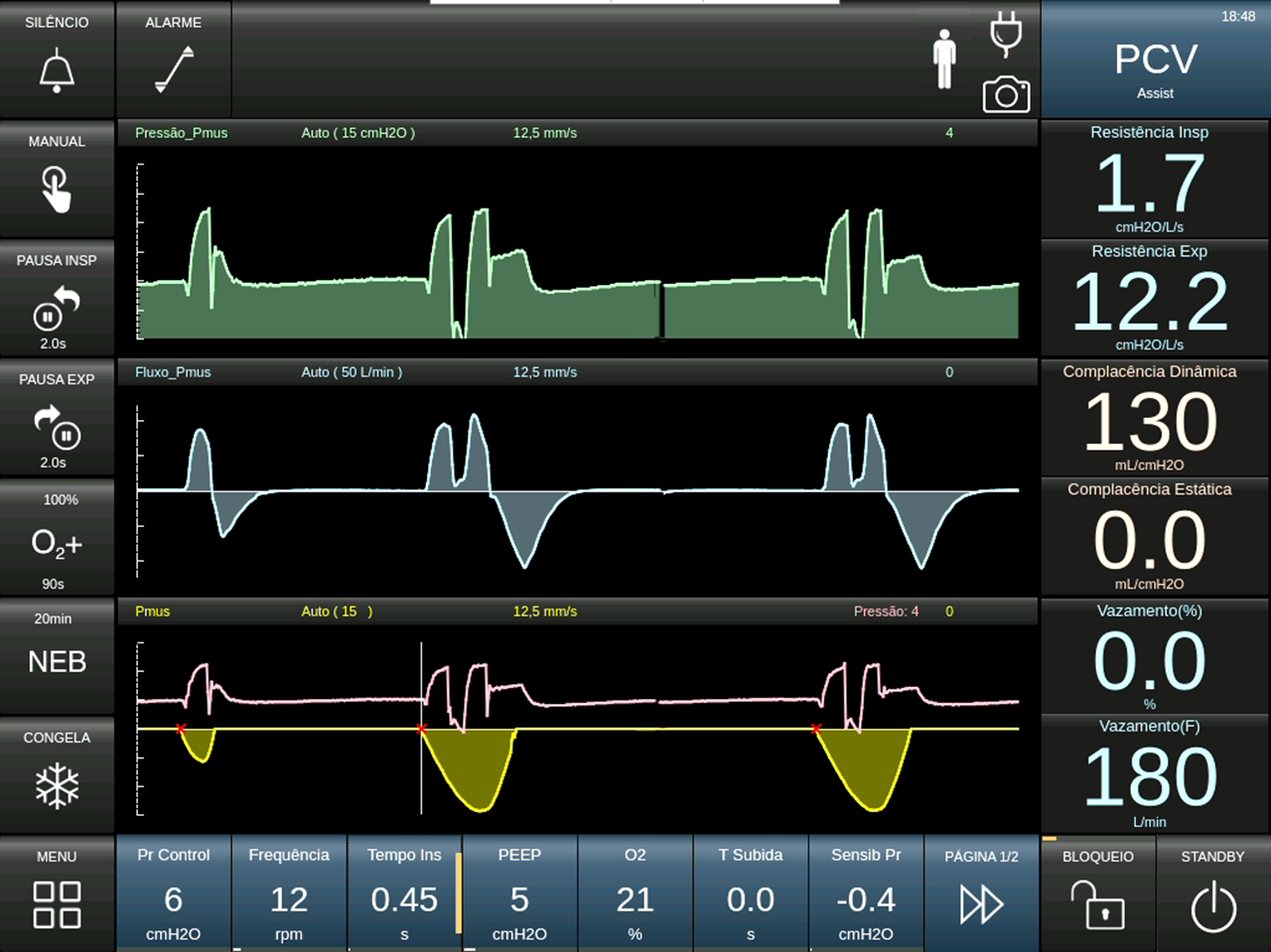  **Control** \|  \| eFigure 8**:** Example of ineffective effort asynchrony. Identical pressure (green) and flow (blue) waveforms were displayed for both groups. In addition to the conventional waveforms, the P_mus_ group could see the estimated inspiratory muscle pressure (yellow) immediately below the airway pressure (pink). Note that one effort was missed after the second ventilator cycle leading to downward deformation in the pressure curve and an upward deformation in the flow curve (arrows). This effort could easily be seen in the P_mus_ curve. \| \| \| --- \| --- \| \| 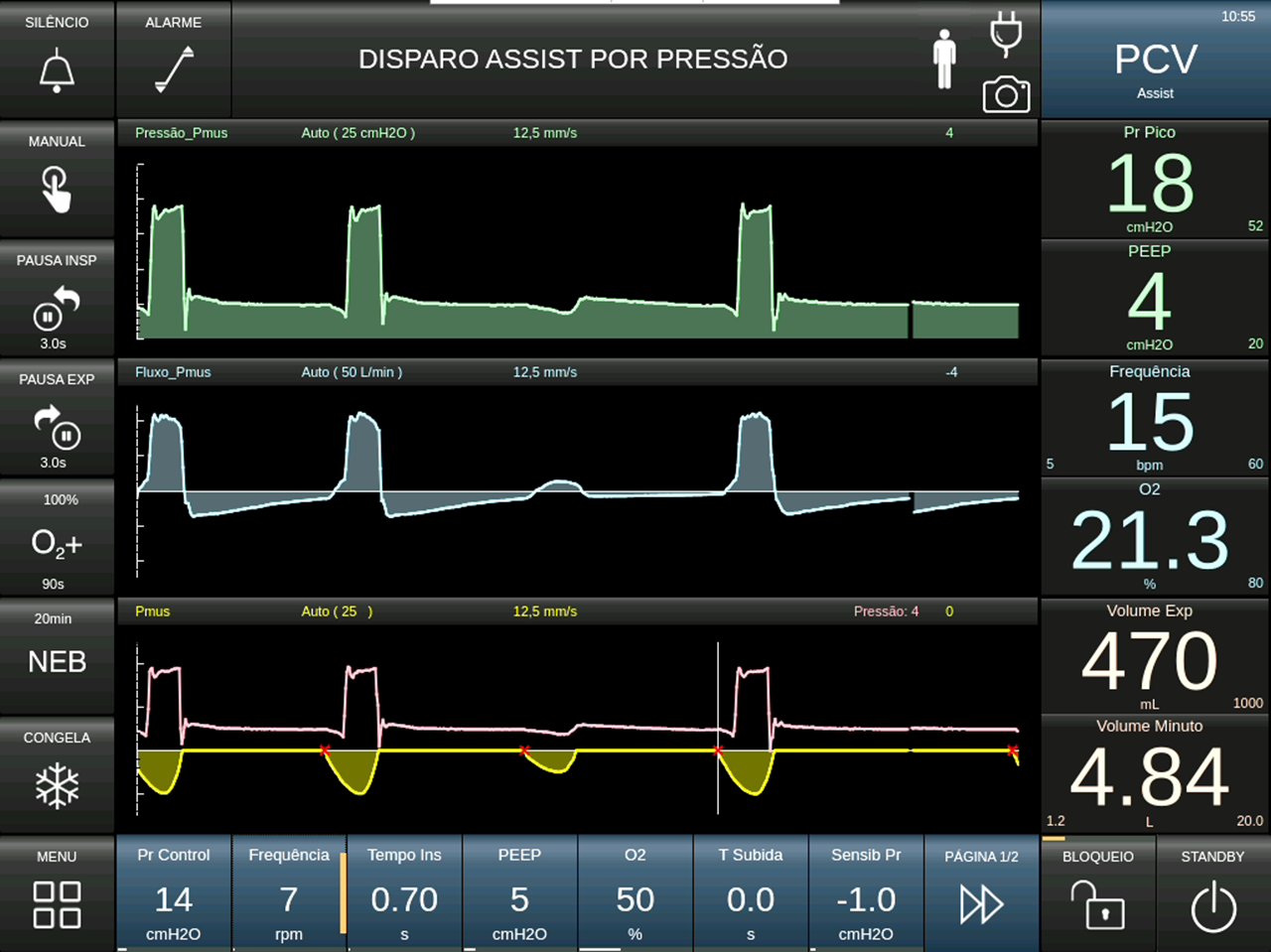  **P_mus_** \| 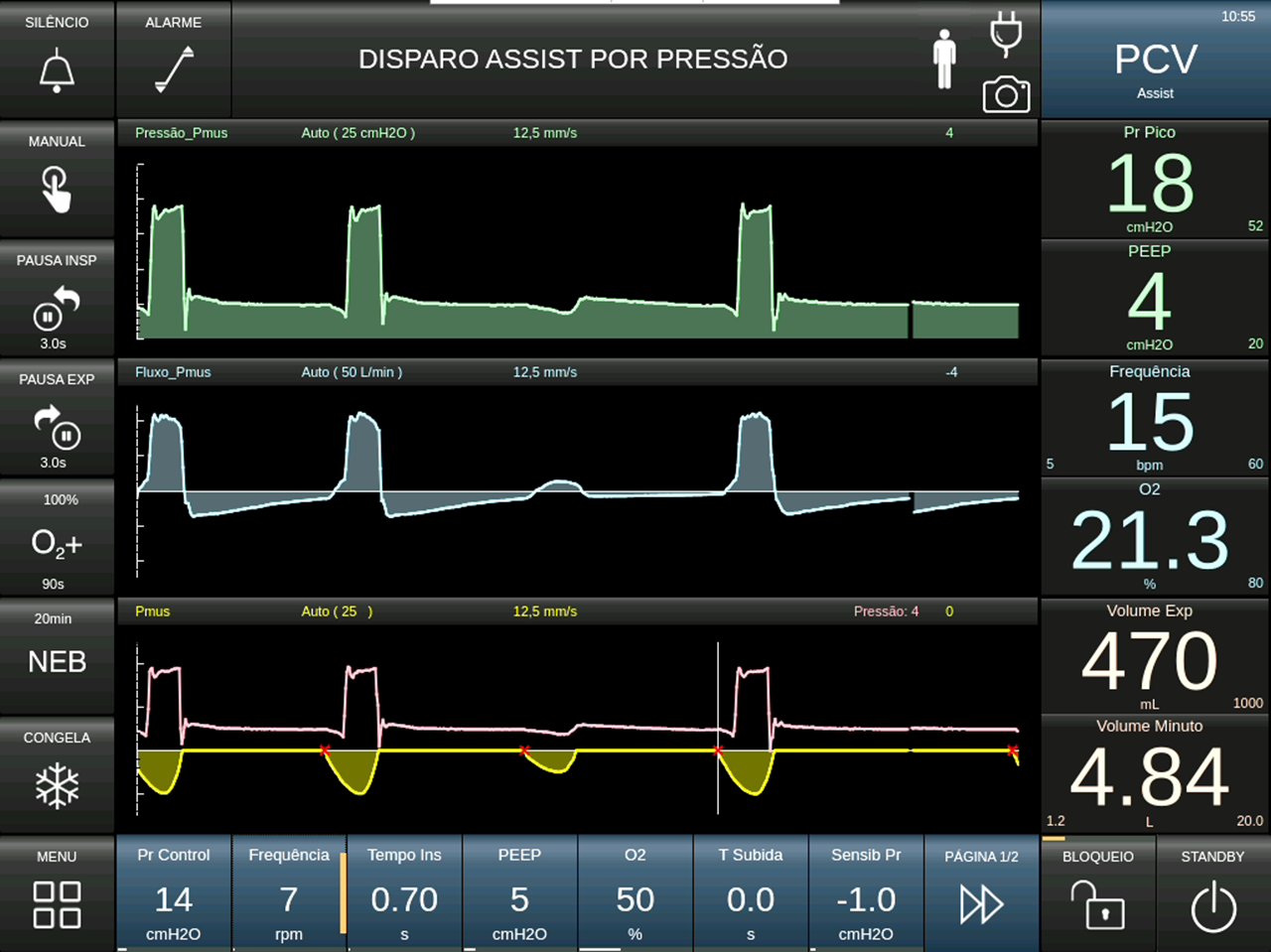  **Control** \|  \| eFigure 9**:** Example of auto-triggering asynchrony. Identical pressure (green) and flow (blue) waveforms were displayed for both groups. In addition to the conventional waveforms, the P_mus_ group could see the estimated inspiratory muscle pressure (yellow) immediately below the airway pressure (pink). Note that in this pressure support mode there are three cycles that were triggered without any inspiratory effort (arrows). These cycles had a shorter inspiratory time and lower peak flows than the adequately triggered cycles. Also note that there was early cycling in the cycles triggered by the inspiratory effort. \| \| \| --- \| --- \| \| 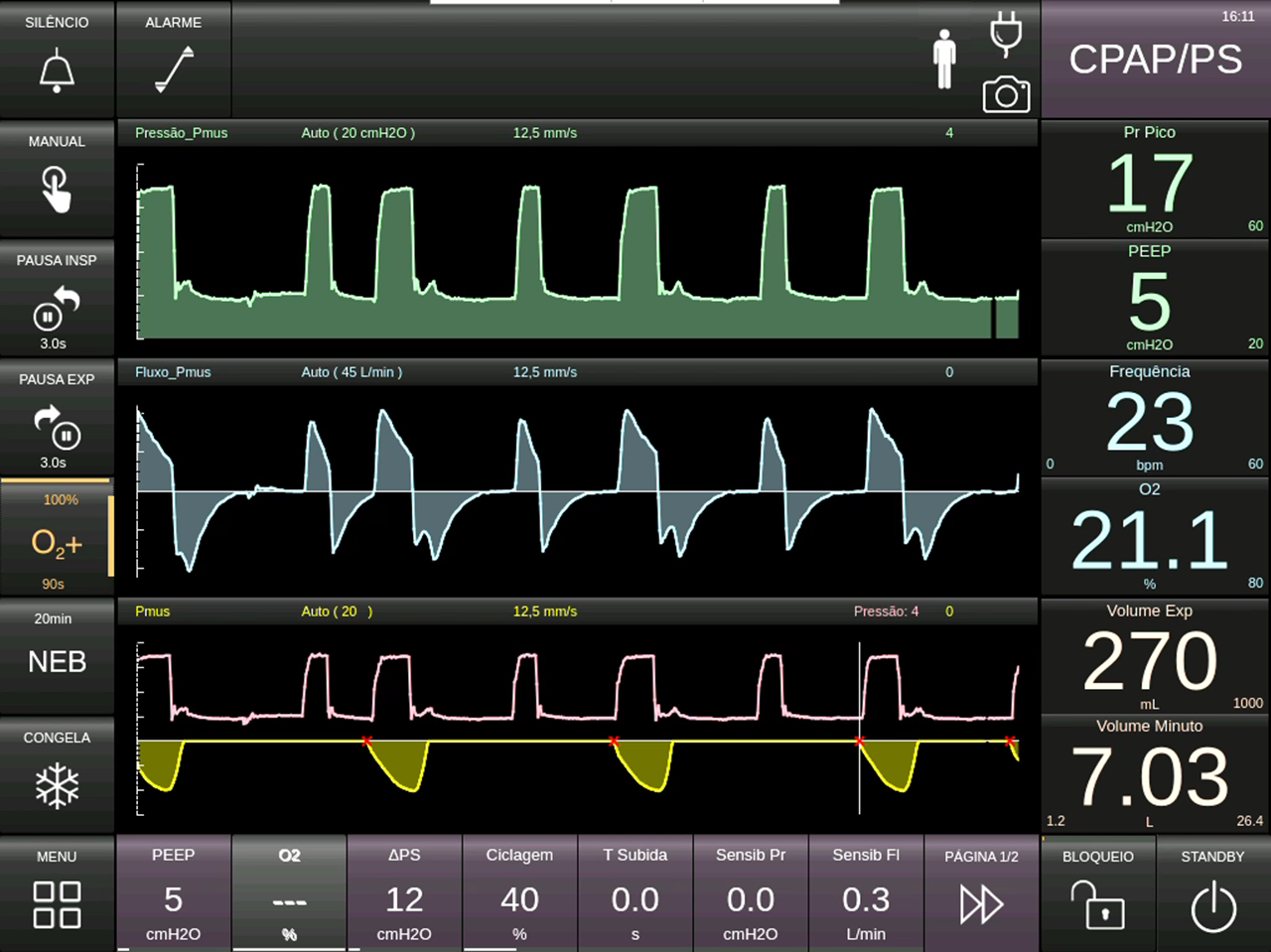  **P_mus_** \| 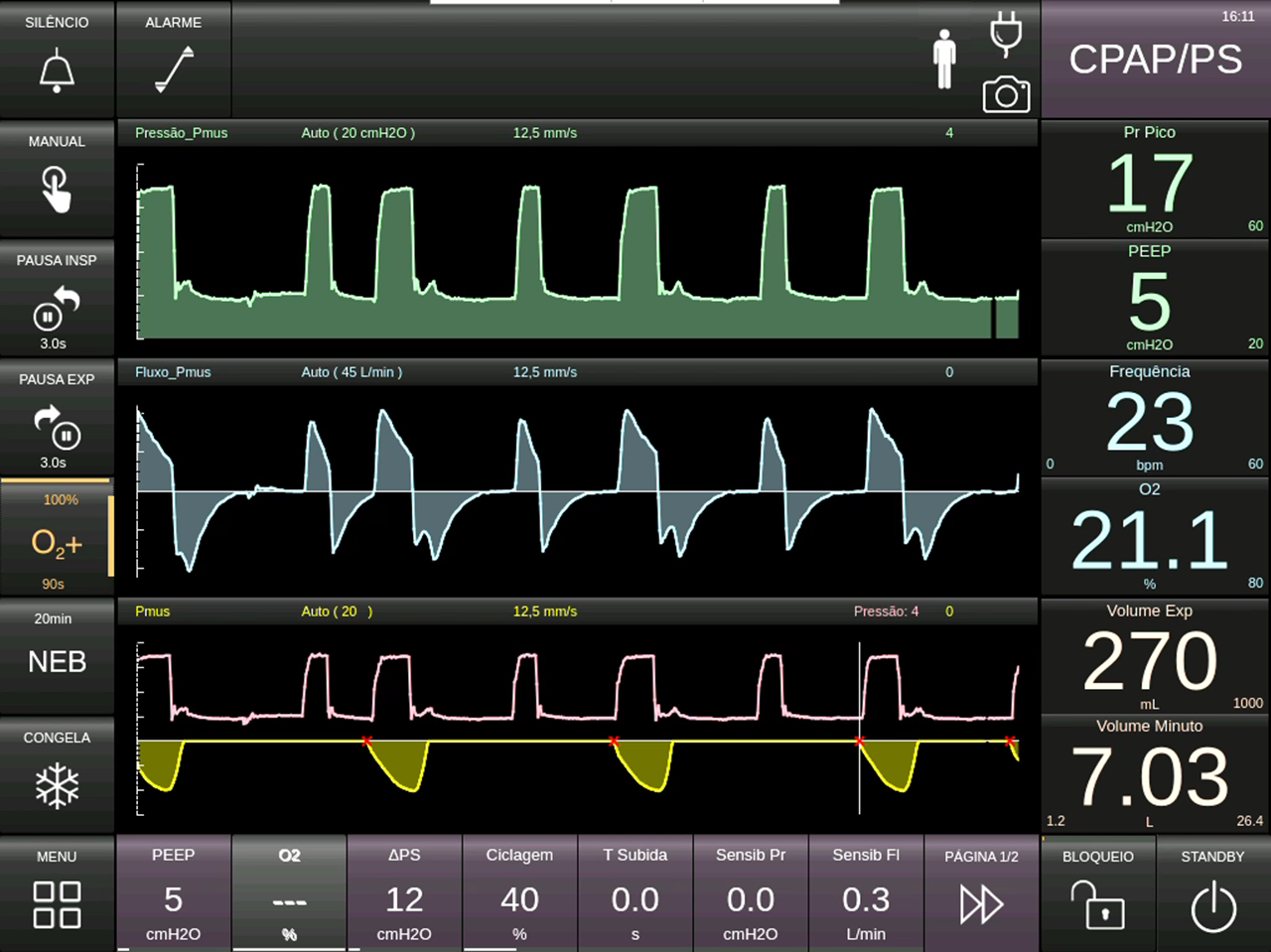  **Control** \|  \| eFigure 10**:** Example of early cycling asynchrony. Identical pressure (green) and flow (blue) waveforms were displayed for both groups. In addition to the conventional waveforms, the P_mus_ group could see the estimated inspiratory muscle pressure (yellow) immediately below the airway pressure (pink). Note the characteristic upward deformation (arrow) in the flow waveform right after cycling-off. The muscle pressure waveform demonstrates that the cycling off occurred before the peak of inspiratory effort. \| \| \| --- \| --- \| \| 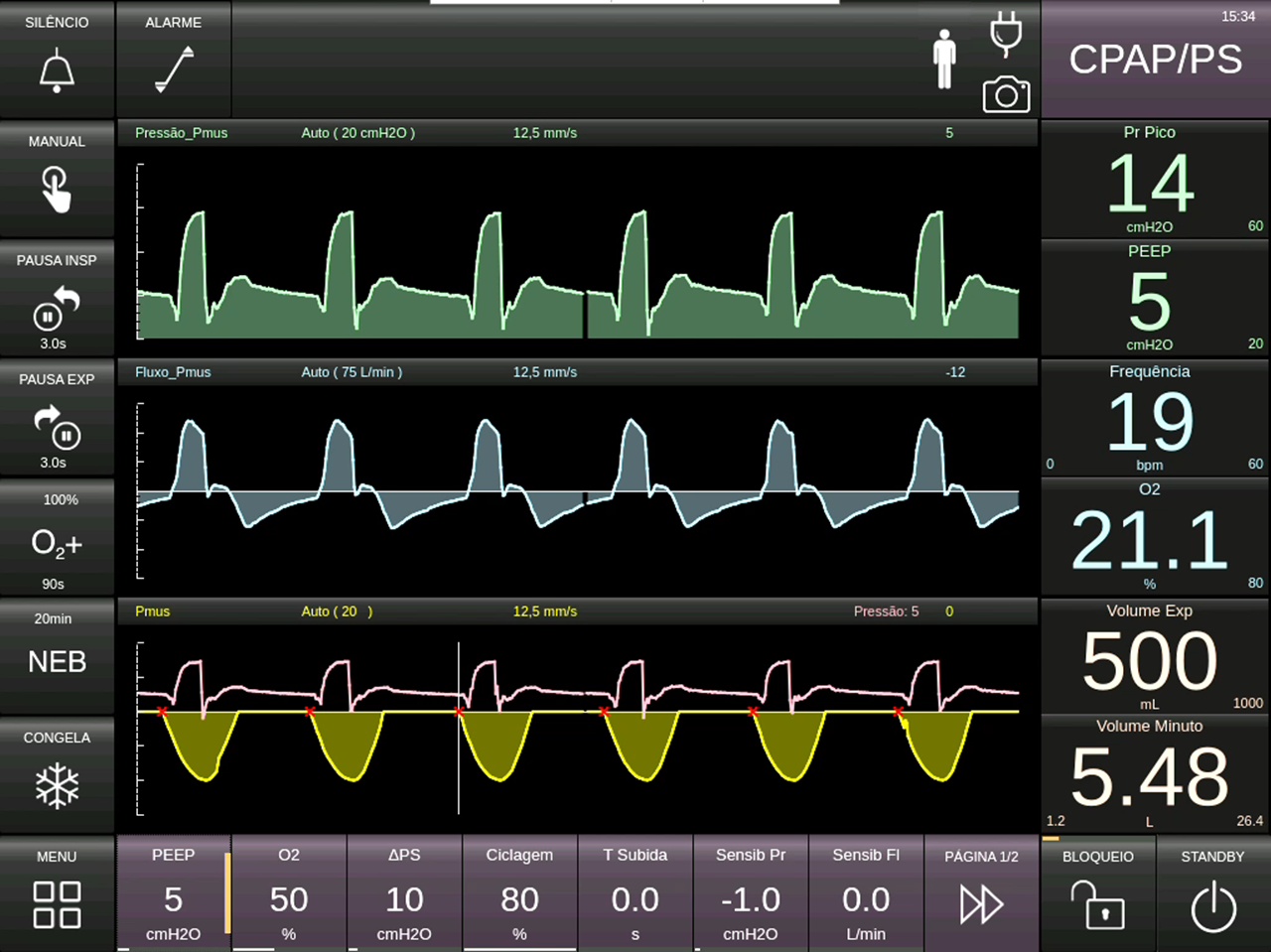  **P_mus_** \| 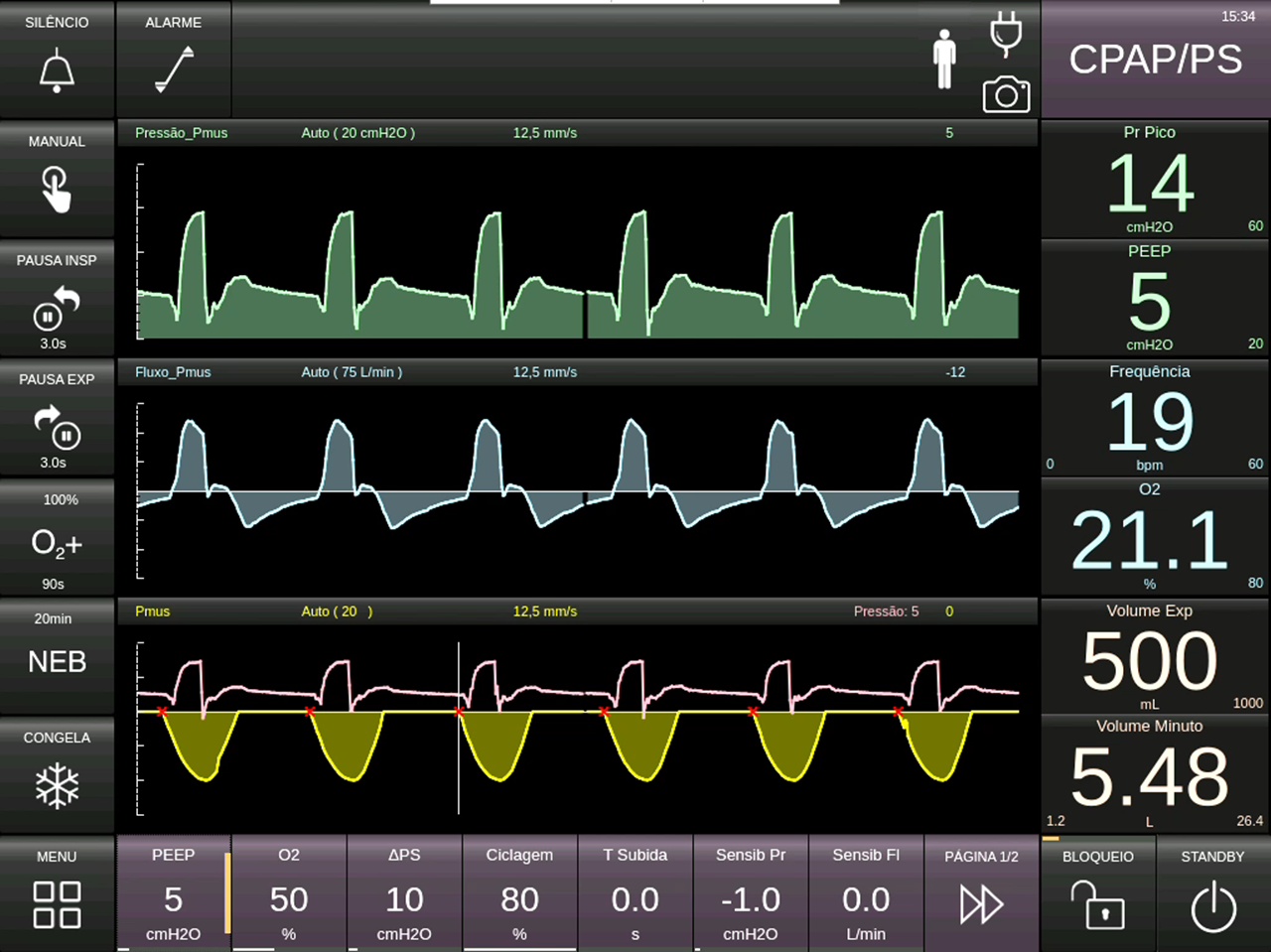  **Control** \|  \| eFigure 11**:** Example of delayed cycling. Identical pressure (green) and flow (blue) waveforms were displayed for both groups. In addition to the conventional waveforms, the P_mus_ group could see the estimated inspiratory muscle pressure (yellow) immediately below the airway pressure (pink). Note the pressure airway pressure overshoot towards the end of inspiration (arrow). This finding is suggestive of delayed cycling-off, a hypothesis that could be easily confirmed by identifying the end of the inspiratory effort during the inspiratory phase. \| \| \| --- \| --- \| \| 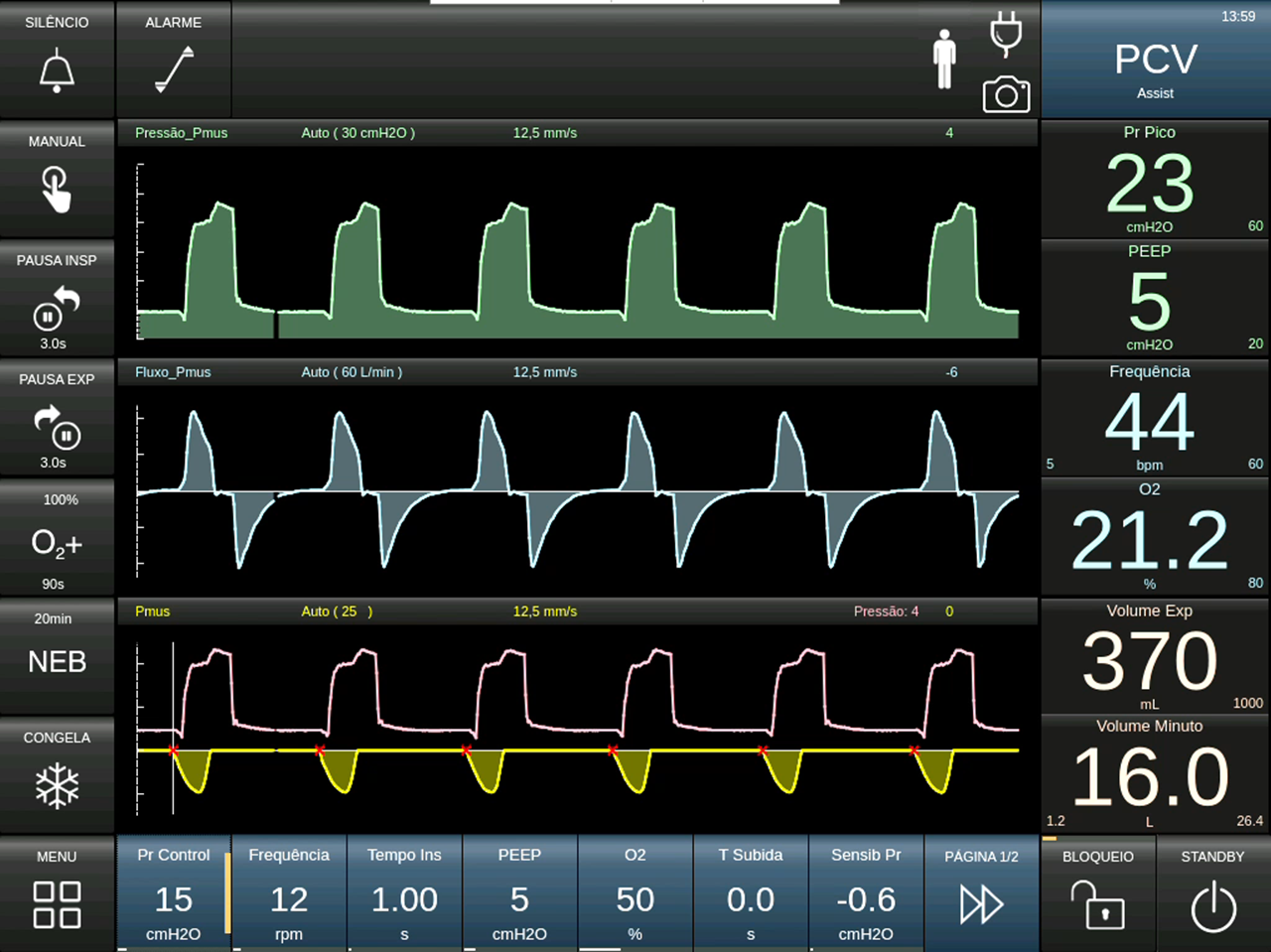  **P_mus_** \| 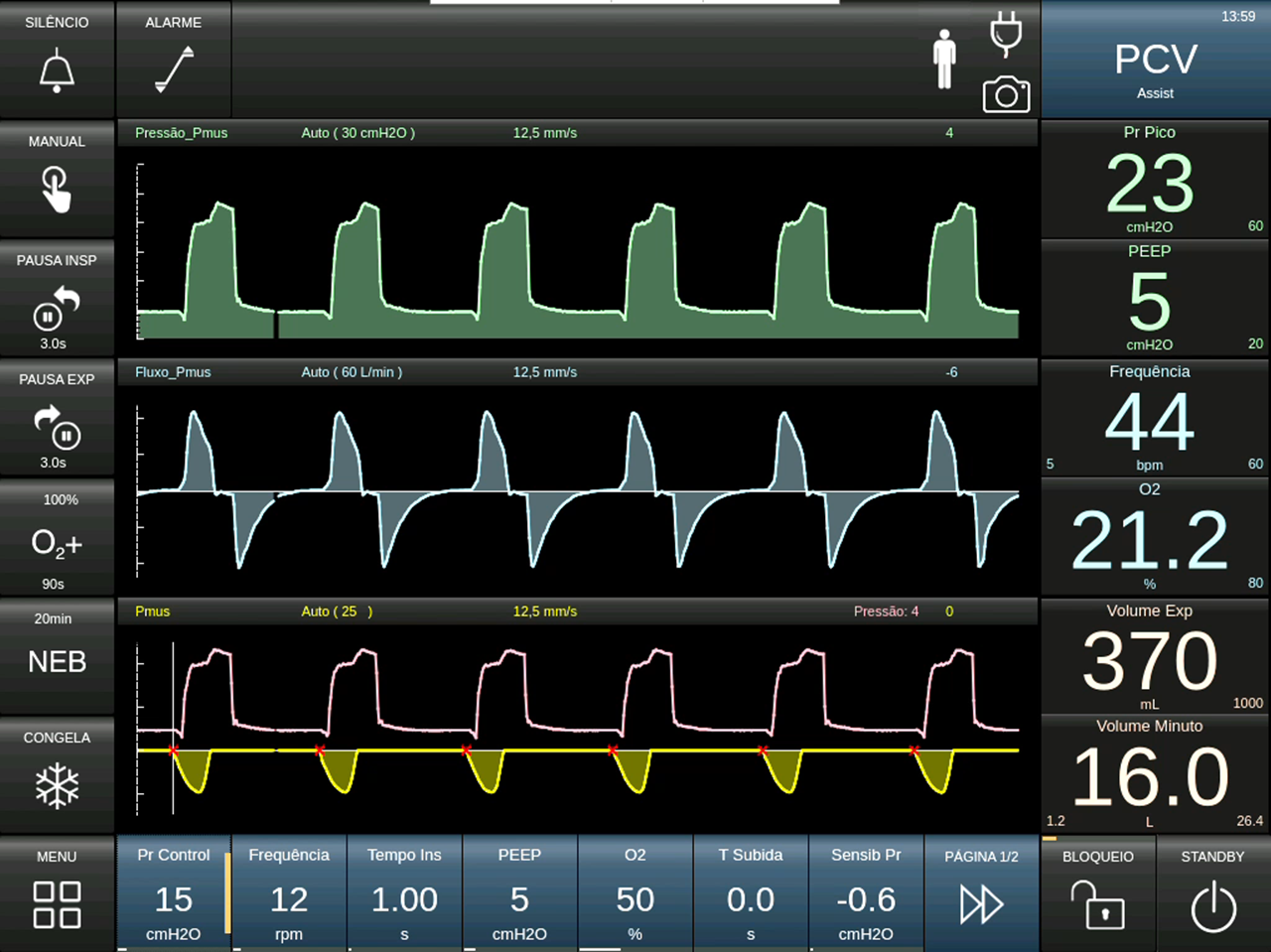  **Control** \| |
| --- | --- | --- | --- | --- | --- | --- | --- | --- | --- | --- | --- | --- | --- | --- | --- | --- | --- | --- | --- | --- | --- | --- | --- | --- |

| eFigure 12**:** Example of delayed cycling. Identical pressure (green) and flow (blue) waveforms were displayed for both groups. In addition to the conventional waveforms, the P_mus_ group could see the estimated inspiratory muscle pressure (yellow) immediately below the airway pressure (pink). This is a more challenging case of late cycling because there is no pressure overshoot. The effort was weak and short ending long before the cycling-off to expiration as easily seen by examining the muscle pressure waveform. The upward concavity of the flow waveform during inspiration is the only cue to identify the delayed cycling. | |
| --- | --- |
| 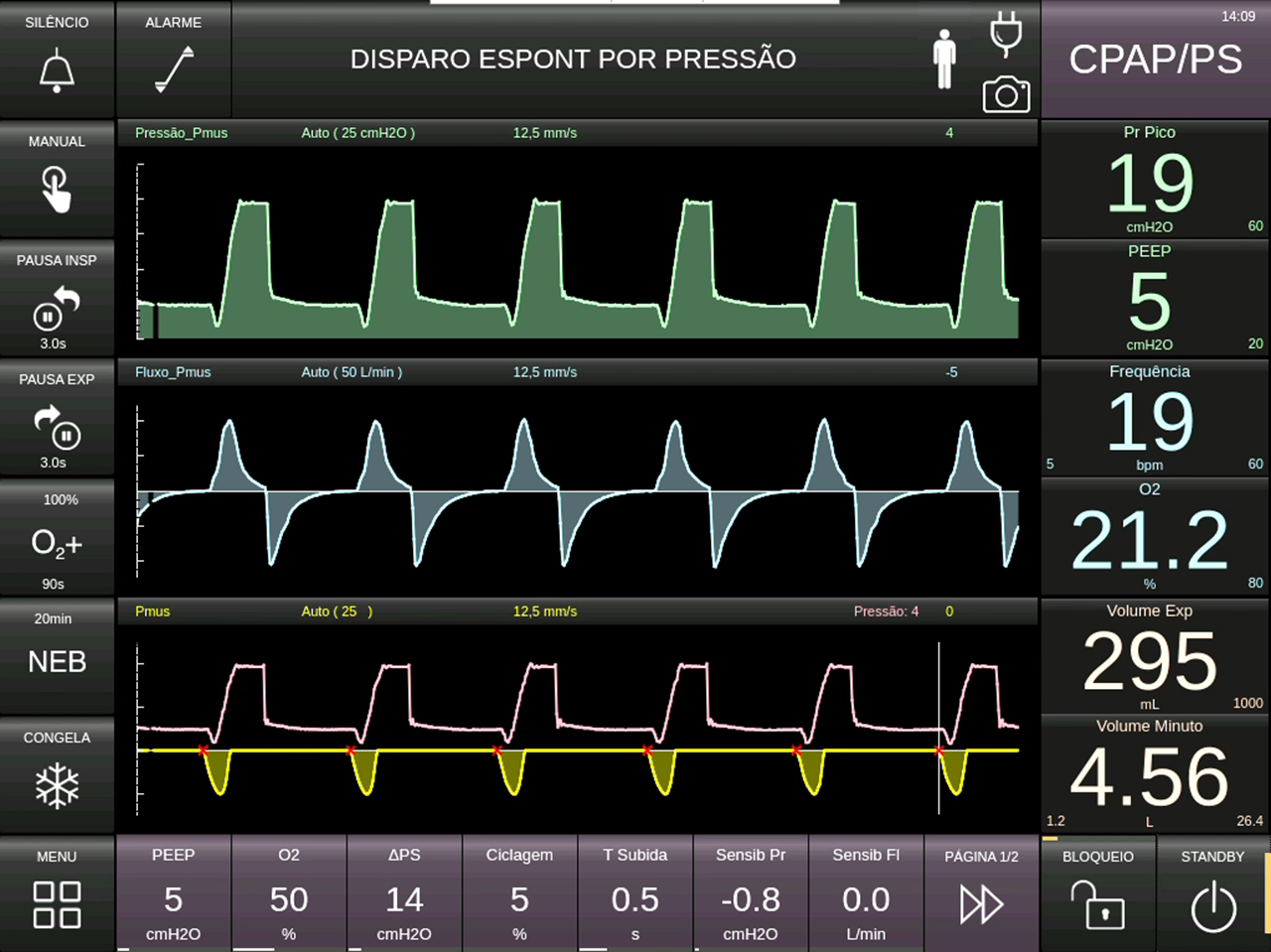  **P_mus_** | 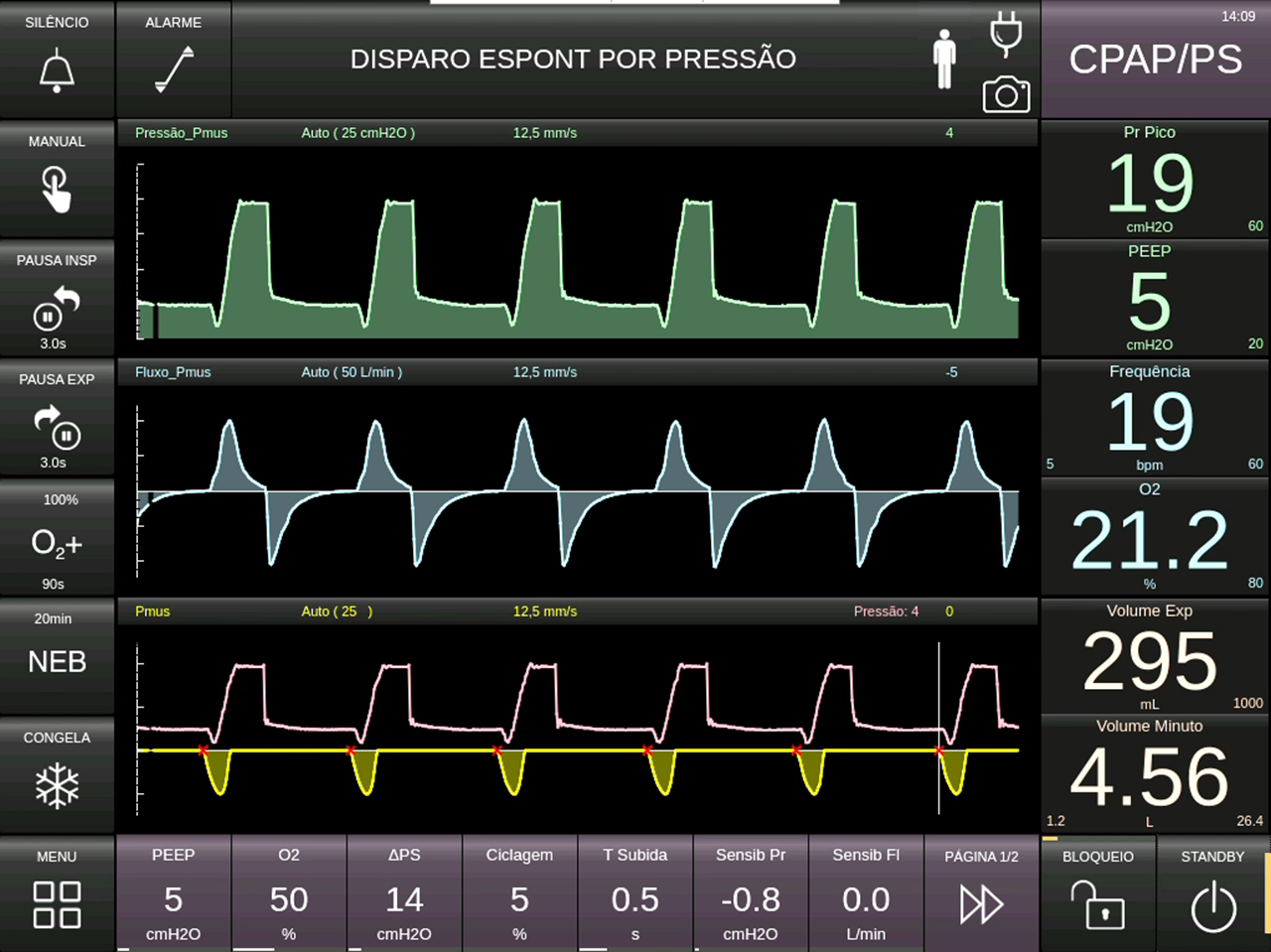  **Control** |

## eTable 1.

ASL 5000 active breathing simulator condition (mechanics of the respiratory system and patient effort) and ventilator parameters ( modality) used per scenario. A total of 49 scenarios were created covering synchronous and asynchronous cycles (ineffective effort, auto-triggering, double-triggering, reverse-triggering, reverse triggering with double cycling, premature cycling, and late cycling).

|  | ASL 5000 | | | |  | Ventilator | | | | | | | | |
| --- | --- | --- | --- | --- | --- | --- | --- | --- | --- | --- | --- | --- | --- | --- |
| Scenario/ Asynchrony | Elastance (cmH2O/L) | Resistance | P_mus_^*^ (cmH_2_O) | RR* |  | Mode | VT (ml) | Pressure (cmH_2_O) | RR | PEEP (cmH_2_O) | Inspiratory time (s) | Trigger^**^ | Flow (L/min) | ET |
| 01/DT | 50 | 10 | 30 | 15 |  | PCV | - | 6 | 12 | 5 | 0.45 | -0.4 cmH2O | - | - |
| 02/IE | 12.5 | 20 | 10/5 | 15 |  | PCV | - | 14 | 7 | 5 | 0.7 | -1.0 cmH2O | - | - |
| 03/DT | 50 | 10 | 15-25 | 15 |  | PSV | - | 14 | - | 5 | - | -0.6 cmH2O | - | 70% |
| 04/DT | 50 | 10 | 30 | 15 |  | PCV | - | 15 | 12 | 5 | 0.65 | -0.6 cmH2O | - | - |
| 05/IE | 20 | 10 | 5-10 | 20 |  | PCV | - | 14 | 10 | 5 | 0.7 | -3 cmH2O | - | - |
| 06/PC | 12.5 | 20 | 5 | 15 |  | VCV | 550 | - | 10 | 5 | - | 0.6 L/min | 60 | - |
| 07/DC | 20 | 10 | 10 | 15 |  | PCV | - | 10 | 7 | 5 | 1.1 | -0.6 cmH2O | - | - |

| 08/RTDT | 20 | 10 | 10-20 | 16-25 |  | VCV | 300 | - | 24 | 5 | - | -0.1 cmH2O | 30 | - |
| --- | --- | --- | --- | --- | --- | --- | --- | --- | --- | --- | --- | --- | --- | --- |
| 9/PC | 20 | 10 | 14 | 20 |  | PSV | - | 10 | - | 5 | - | -1.0 cmH2O | - | 80% |
| 10/AT-PC^***^ | 50 | 10 | 10 | - |  | PSV | - | 12 | - | 5 | - | 09 L/min | - | 40% |
| 11/DC | 50 | 10 | 10 | 20 |  | PCV | - | 15 | 12 | 5 | 1.0 | -0.6 cmH2O | - | - |
| 12/PC | 20 | 10 | 10 | 15 |  | VCV | 400 | - | 10 | 5 | - | -0.6 cmH2O | 45 | - |
| 13/AT-RT^***^ | 50 | 10 | 10 | - |  | PSV | - | 12 | - | 5 | - | 0.9 L/min | - | 5% |
| 14/PC | 20 | 10 | 10 | 15 |  | PCV | - | 15 | 10 | 5 | 0.65 | -1.0 cmH2O | - | - |
| 15/PC-DT^***^ | 12.5 | 20 | 10 | 12 |  | PCV | - | 12 | 10 | 5 | 0.7 | 0.1 L/min | - | - |
| 16/DC | 50 | 10 | 10 | 20 |  | PSV | - | 14 | - | 5 | - | -0.8 cmH2O | - | 5% |
| 17/IE-DC^***^ | 12.5 | 20 | 10 | 35 |  | PSV | - | 16 | - | 5 | - | -2 cmH2O | - | 60% |
| 18/DT | 20 | 10 | 5-10 | 15 |  | VCV | 350 | - | 12 | 5 | - | -0.2 cmH2O | 55 | - |
| 19/DT | 20 | 10 | 30 | 6 |  | PCV | - | 15 | 12 | 5 | 0.85 | -0.6 cmH2O | - | - |
| 20/PC | 50 | 10 | 10 | 25 |  | PCV | - | 15 | 10 | 5 | 0.7 | 1 L/min | - | - |
| 21/RT-DC^***^ | 20 | 10 | 8-10 | 13-18 |  | VCV | 500 | - | 15 | 5 | - | -1.6 cmH2O | 50 | - |
| 22/PC | 50 | 10 | 10 | 25 |  | VCV | 250 | - | 10 | 5 | - | -1.0 cmH2O | 40 | - |
| 23/RTDT | 20 | 10 | 8-15 | 13-16 |  | PCV | - | 10 | 15 | 5 | 0.7 | -0.4 cmH2O | - | - |
| 24/S | 12.5 | 20 | 10 | 12 |  | PSV | - | 8 | - | 5 | - | -0.4 cmH2O | - | 80% |
| 25/RT | 20 | 10 | 8-10 | 13-18 |  | PCV | - | 10 | 15 | 5 | 0.7 | -1.2 cmH2O | - | - |
| 26/IE-DC^***^ | 20 | 10 | 5-7 | 20 |  | VCV | 400 | - | 10 | 5 | - | -1.8 cmH2O | 50 | - |
| 27/RT-DC^***^ | 20 | 10 | 8/-10 | 15-18 |  | VCV | 450 | - | 16 | 5 | - | -1.2 cmH2O | 60 | - |
| 28/PC | 50 | 10 | 10 | 25 |  | PSV | - | 15 | - | 5 | - | -0.6 cmH2O | - | 50% |
| 29/RT-DC^***^ | 12.5 | 20 | 10 | 13-18 |  | VCV | 400 | - | 15 | 5 | - | -1.6 cmH2O | 40 | - |
| 30/IE-DC^***^ | 50 | 10 | 2-15 | 20 |  | PSV | - | 14 | - | 5 | - | -0.6 cmH2O | - | 25% |
| 31/RTDT^***^ | 12.5 | 20 | 5-20 | 11-14 |  | VCV | 350 | - | 12 | 5 | - | -0.2 cmH2O | 55 | - |
| 32/DT | 50 | 10 | 20 | 15 |  | VCV | 300 | - | 10 | 5 | - | -0.4 cmH2O | 50 | - |
| 33/IE-DC | 12.5 | 20 | 5-10 | 24 |  | VCV | 500 | - | 10 | 5 | - | -1.4 cmH2O | 50 | - |
| 34/PC | 12.5 | 20 | 10 | 12 |  | PSV | - | 10 | - | 5 | - | 0.6 L/min | - | 80% |
| 35/IE-DT^***^ | 12.5 | 20 | 8-30 | 15 |  | PSV | - | 14 | - | 5 | - | -0.4 cmH2O | - | 80% |
| 36/RTDT | 50 | 10 | 10-20 | 16-25 |  | VCV | 400 | - | 15 | 5 | - | -0.6 cmH2O | 30 | - |
| 37/S | 50 | 10 | 12 | 25 |  | PSV | - | 8 | - | 5 | - | -1.0 cmH2O | - | 55% |
| 38/DT-PC^***^ | 20 | 10 | 15-30 | 15 |  | PSV | - | 15 | - | 5 | - | 0.1 L/min | - | 80% |
| 39/IE-DC^***^ | 50 | 10 | 5-10 | 24 |  | PCV | - | 14 | 11 | 5 | 0.7 | -1.6 cmH2O | - | - |
| 40/DT | 12.5 | 10 | 20 | 5 |  | PCV | - | 15 | 15 | 5 | 0.7 | -0.6 cmH2O | - | - |
| 41/S | 20 | 10 | 15 | 16 |  | PCV | - | 15 | 15 | 5 | 0.55 | 0.4 cmH2O | - | - |
| 42/IE-DC^***^ | 20 | 10 | 3-5 | 20 |  | PSV | - | 14 | - | 5 | - | -1.6 cmH2O | - | 25% |
| 43/DT | 12.5 | 20 | 20 | 5 |  | VCV | 500 | - | 15 | 5 | - | -0.6 cmH2O | 45 | - |
| 44/IE-DC^***^ | 50 | 10 | 10 | 38 |  | PSV | - | 17 | - | 5 | - | -1.6 cmH2O | - | 5% |
| 45/S | 20 | 10 | 10 | 18 |  | PCV | - | 15 | 15 | 5 | 0.55 | -0.4 cmH2O | - | - |
| 46/DT | 50 | 10 | 30 | 15 |  | PCV | - | 8 | 10 | 5 | 0.45 | -0.6 cmH2O | - | - |
| 47/IE-DC^***^ | 20 | 10 | 10 | 40 |  | PSV | - | 14 | - | 5 | - | -1.6 cmH2O | - | 25% |
| 48/RT-DC^***^ | 50 | 10 | 8-10 | 13-18 |  | VCV | 500 | - | 15 | 5 | - | -1.6 cmH2O | 50 | - |
| 49/IE-DC^***^ | 50 | 10 | 5-7 | 24 |  | VCV | 300 | - | 10 | 5 | - | -2 cmH2O | 45 | - |

*In some scenarios a different combination of respiratory rate and inspiratory muscle pressure was used to achieve the expected asynchrony.

** Pressure or flow triggers were used per scenario.

*** Two asynchronies in the same scenario.

P_mus_: Inspiratory Muscle Pressure. RR: respiratory rate; VT: tidal volume; Pressure: pressure adjusted on the mechanical ventilation; ET: expiratory trigger; PCV: Pressure controlled ventilation; VCV: Volume controlled ventilation; PSV: Pressure support ventilation; PEEP: Positive end expiratory pressure; DT: Double-Triggering; IE: Ineffective effort; PC: Premature Cycling; DC: Delayed Cycling; RT: Reverse Triggering; RTDC: Reverse triggering with double cycling; AT: Auto-Triggering; S: Syncronous.

| eFigure 13: Asynchrony detection rate (sensitivity) stratified according to asynchrony type in the inspiratory muscle pressure (Pmus) group as compared to the control group. Error bars represent the standard error of the mean. |
| --- |
| 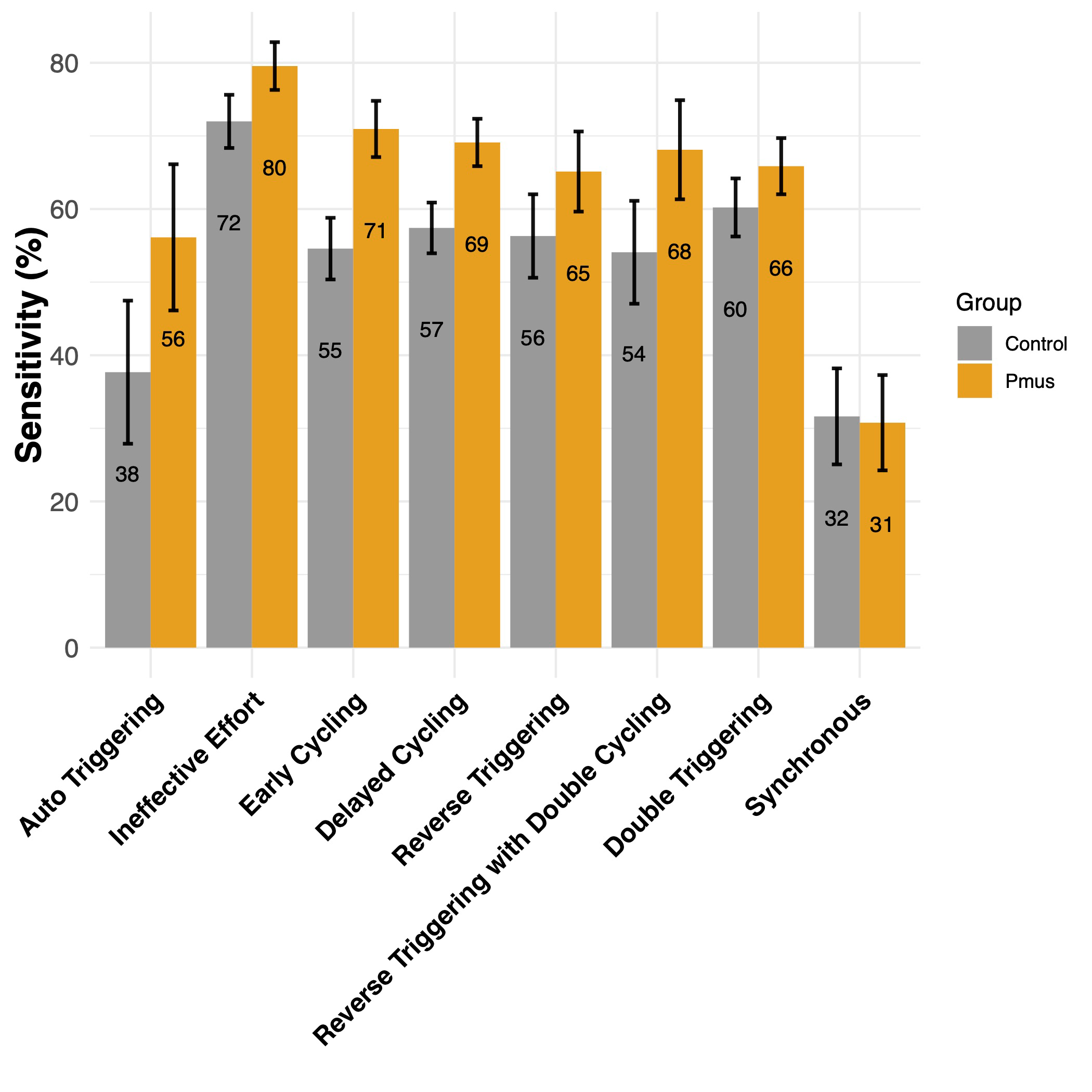 |

| eFigure 14: Specificity of asynchrony detection stratified according to asynchrony type in the inspiratory muscle pressure (P_mus_) group as compared to the control group. Error bars represent the standard error of the mean. |
| --- |
| 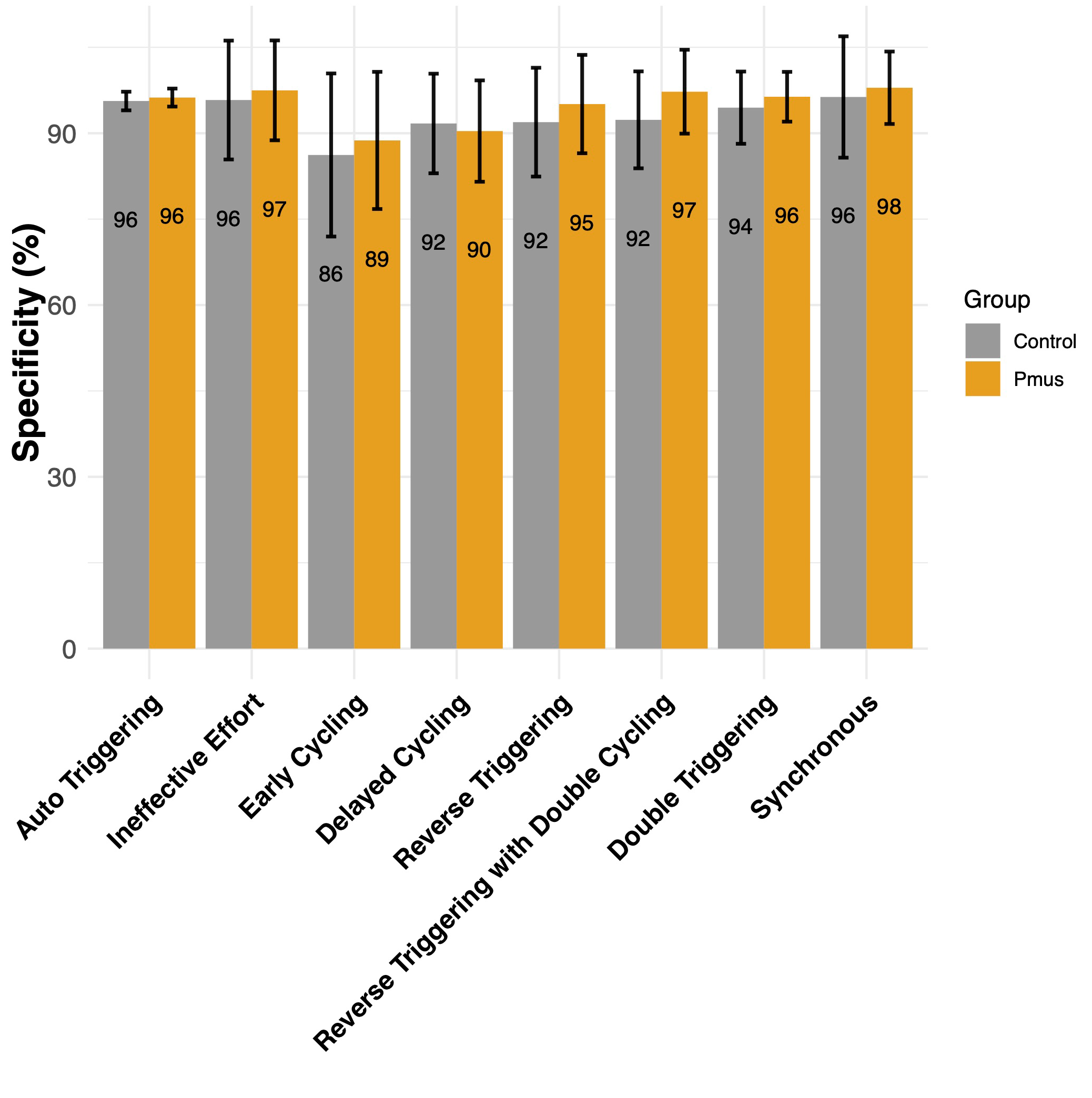 |

# References

1. Schuster M, Paliwal K. Bidirectional Recurrent Neural Networks. EEE TRANSACTIONS ON SIGNAL PROCESSING, 1997. VOL. 45, NO. 11. https://maxwell.ict.griffith.edu.au/spl/publications/papers/ieeesp97_schuster.pdf
2. How to Develop a Bidirectional LSTM For Sequence Classification in Python with Keras - https://machinelearningmastery.com/develop-bidirectional-lstm-sequence-classification-python-keras/
3. A Beginner's Guide to LSTMs and Recurrent Neural Networks - https://wiki.pathmind.com/lstm
4. Understanding LSTM Networks - <http://colah.github.io/posts/2015-08-Understanding-LSTMs/>
5. Understanding LSTM and its diagrams - https://medium.com/mlreview/understanding-lstm-and-its- diagrams-37e2f46f1714
